# Development and validation of the MosquitoWise survey to assess perceptions towards mosquitoes and mosquito-borne viruses in Europe

**DOI:** 10.1038/s41598-024-52219-9

**Published:** 2024-01-20

**Authors:** Ayat Abourashed, Pauline A. de Best, Laura Doornekamp, Reina S. Sikkema, Eric C. M. van Gorp, Aura Timen, Frederic Bartumeus, John R. B. Palmer, Marion P. G. Koopmans

**Affiliations:** 1 Viroscience, Erasmus University Medical Center, Rotterdam, 3015 GD The Netherlands; 2 Centre d’Estudis Avançats de Blanes (CEAB-CSIC), 17300 Blanes, Spain; 3 National Institute for Public Health and the Environment (RIVM), Bilthoven, 3721 MA The Netherlands; 4 Department of Medical Microbiology and Infectious Diseases, University Medical Center, Rotterdam, 3015 GD The Netherlands; 5 Centre for Avian Migration, Netherlands Institute of Ecology (NIOO-KNAW), Wageningen, 6708 PB The Netherlands; 6 Department of Primary and Community Care, RadboudUMC, Nijmegen, 6525 GA The Netherlands; 7 Athena Institute, VU University, Amsterdam, 1081 HV The Netherlands; 8 Centre de Recerca Ecològica i Aplicacions Forestals (CREAF), 08193 Cerdanyola del Vallès, Spain; 9 Institució Catalana de Recerca i Estudis Avançats (ICREA), 08010 Barcelona, Spain; 10 Department of Political and Social Sciences, Universitat Pompeu Fabra, 08005 Barcelona, Spain

**Keywords:** Public health, Infectious diseases

## Abstract

Due to climate change and the expanding geographical ranges of key mosquito species, several mosquito-borne viruses (MBVs) have recently emerged in Europe. Understanding people’s perceptions and behaviours towards these viruses and the mosquitoes capable of transmitting them is crucial for implementing effective prevention measures and targeted communication campaigns. However, there is currently no appropriate validated survey for European populations to assess this. This study developed and validated a standardized survey, based on the Health Belief Model (HBM), to assess perceptions of mosquitoes and MBVs among Europe’s residents. The survey was distributed online to United Kingdom (UK), Dutch and Spanish participants through panel providers. Survey validity and reliability were tested using confirmatory factor analysis (CFA) and Cronbach’s alpha. The optimised survey was completed by 336 UK, 438 Dutch and 475 Spanish residents, respectively, and the HBM items passed our validity and reliability testing in all three countries. The final survey has 57 questions, including 19 validated HBM items, and questions to assess demographic characteristics, knowledge, prevention measures and behavioural determinants. Our MosquitoWise survey bridges researchers' understandings of European residents’ perceptions and knowledge as a first step to improve preventive behaviour towards mosquitoes and MBVs and guide prevention and communication initiatives.

## Introduction

In recent years, several MBVs have emerged in Europe, resulting in autochthonous transmission caused by both invasive and local mosquito species. While most mosquito-borne virus cases (MBVs) occur in Africa and Southeast Asia, increased changes in climate and land use in combination with the expanding geographical ranges of key invasive mosquito species *Aedes aegypti* and *Aedes albopictus* drive the potential for more mosquito-borne arboviruses to become endemic in Europe^1,2^. International trade and human movement have proven to be crucial drivers of the spread of invasive mosquitoes, with eggs and adult mosquitoes transported in shipping containers, cars and other vehicles^3^. This phenomenon is well depicted by the introduction of *Ae. albopictus* in Spain, where the first *Ae. albopictus* was found in 2004 and believed to have arrived in a shipment of used tires^4^. Since then, this species has been regularly detected along the Mediterranean coast of Spain^5^. While it is known as a nuisance, *Ae. albopictus* is a primary vector for dengue and chikungunya viruses. These growing *Ae. albopictus* populations across Europe raises risks for autochthonous viral transmission within human populations^1^.

Following the arrival of *Ae. albopictus* in Europe, local outbreaks of dengue and chikungunya have been reported in Croatia, France, Italy, Portugal, Spain and more^1,2,6^. In 2022, 71 cases of locally acquired dengue were reported in mainland Europe (65 in France and 6 in Spain), matching the cumulative count of cases reported from 2010 to 2021^6^. Other emerging mosquito-borne viruses like West Nile virus (WNV) have been spread mainly by *Culex pipiens*, a native mosquito species found almost everywhere in Europe^1^. WNV outbreaks regularly occur in Greece and Italy, and new autochthonous cases in humans have been detected as far north as the Netherlands in 2020^1,6,7^. In the EU/EEA, 1133 human WNV cases were reported in 2022. Of these cases, there were 92 deaths and 1112 locally acquired infections, making this a record number of cases since 2018^6^. With this increase in local MBV prevalence, Europe’s residents are facing a new public health threat.

One of the key public health measures to reduce MBV infections is to take effective prevention measures against mosquito bites^8^. Successful implementation of such measures greatly depends on the knowledge and behaviour of the general public. Establishing the basic knowledge and beliefs people have regarding mosquitoes and the viruses they can potentially transmit is an important step in designing effective communication strategies^9^. Questionnaires that assess perceptions, knowledge and behaviour of people towards mosquitoes and MBVs have been widely used but focus on residents of endemic countries in the Americas, South-East Asia and the Western Pacific regions^10–16^. The few questionnaires that have been developed for European populations have focused on either invasive mosquitoes or specific MBVs^17–21^. Currently, there is no appropriate survey validated for European populations that assesses perceptions, knowledge, and behaviour towards prevention measure use for mosquitoes and MBVs in general.

There are many models of human behaviour that can guide survey development in this area. One that is well-established, and particularly relevant, is the Health Belief Model (HBM). The HBM was specifically developed to study people’s perceptions of health risks and influences of these perceptions on their decision to engage in preventive behaviour to promote their health. The model aims to measure certain ideas or concepts, also known as constructs, to assess intent to use preventive behaviours. Since constructs are not directly observable, a group of items can be used to infer what the construct is aiming to measure^22^. The model includes six constructs (Perceived Susceptibility, Perceived Severity, Perceived Benefits, Perceived Barriers, Cues to Action and Self-Efficacy) which altogether help predict people’s behaviours^23,24^. Initially designed to explain the adoption of preventive health behaviours in the United States, the HBM has been adapted for various contexts and topics^25^.

As MBV risk differs across Europe, a survey that can capture these differences in exposure and their effects on perceptions of health risks is particularly needed but did not yet exist prior to this study. We use the HBM to develop and validate such a survey, suitable for Europe-wide implementation. Furthermore, although the HBM does not account for knowledge as an influence on behaviour, our survey includes items to measure knowledge, along with demographic characteristics, attitudes toward prevention measures and other potential behavioural determinants.

Here, we present the design, validation and translations of the MosquitoWise survey in three European countries.

## Methods

**Figure 1:**
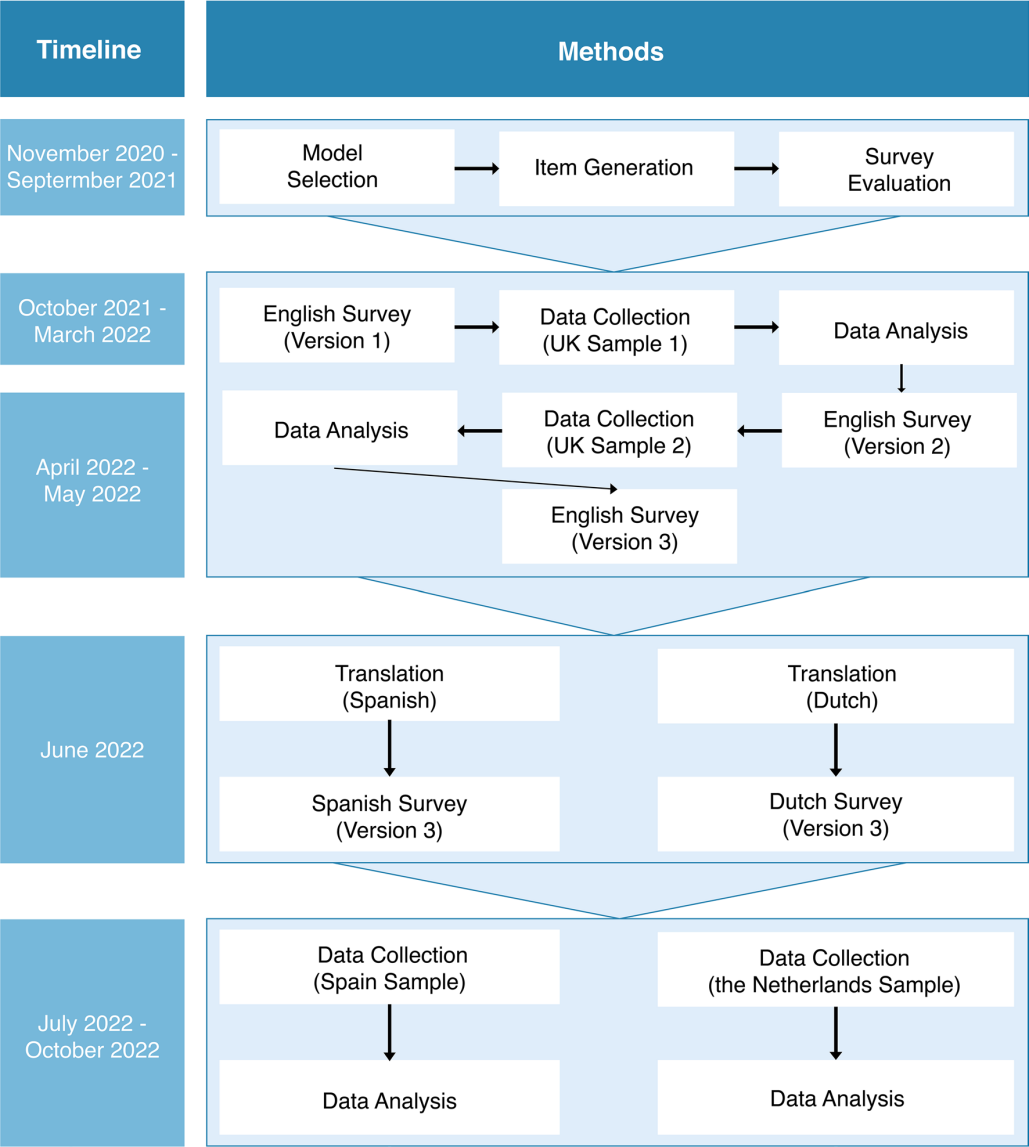
Complete overview and timeline of development, translations, data collection and data analysis for validity assessment of the English, Dutch and Spanish survey versions.

The development and validation of this MosquitoWise survey were based on previously published guidelines on this process^26^. Using the methods described by Boateng et al., we address the development of questions, hereafter called items, and the survey’s validity in three languages. Our study methods are described in detail below, divided into three main parts: survey development and translation, data collection using a representative population panel to gather participants’ survey responses, and data analysis for validity assessment of the survey using participant responses (Fig. 1).

### Survey development and translation

#### Target populations

We developed the survey to capture perceptions, knowledge and behaviour in European populations with different mosquito abundances, as well as different mosquito-borne viral prevalence The survey was distributed among the general populations, aged 18 and over, of three countries: the United Kingdom, the Netherlands and Spain^22,27–29^.

#### Model selection and item generation

**Figure 2:**
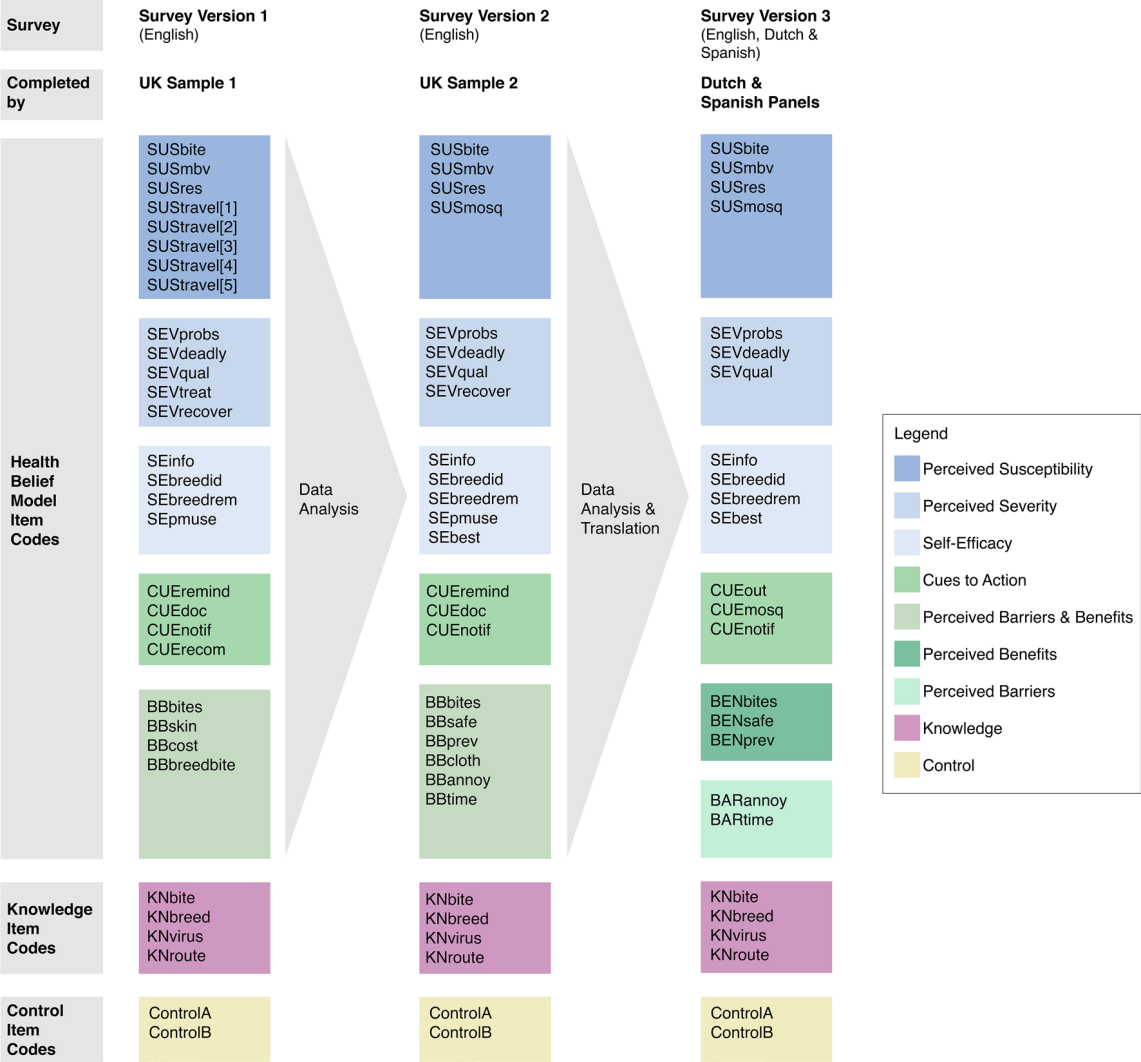
Overview of item codes of all three survey versions. Each code represents one item in the surveys. All the Health Belief Model items follow a 7-point Likert scale*. UK* United Kingdom, *SUS* Perceived Susceptibility, *SEV* Perceived Severity, *SE* Self-Efficacy, *CUE* Cues to Action, *BB* Perceived Barriers and Benefits (combined), *BEN* Perceived Benefits, *BAR* Perceived Barriers, *KN* Knowledge.

The survey was structured based on the HBM, which matches this study’s research aims^23,24^. Our survey included the following HBM constructs: Perceived Susceptibility (SUS), Perceived Severity (SEV), Perceived Barriers (BAR), Perceived Benefits (BEN), Self-Efficacy (SE) and Cues to Action (CUE) (Fig. 2). Based on these constructs, items were developed in collaboration with several virology, entomology and behavioural experts. Items were measured with a 7-point Likert scale, which is commonly used for participants to rate their level of agreement or disagreement with a statement^30^. Each Item is rated as 1 (Strongly Disagree), 2 (Disagree), 3 (Somewhat Disagree), 4 (Neutral), 5 (Somewhat Agree), 6 (Agree) And 7 (Strongly Agree). Additional items (specifically on knowledge, prevention measure use, and demographics) were included to gauge behaviour. Two control items (such as “Please select ‘Somewhat Disagree’ as your answer choice”) were added to check if participants completed the survey with authentic responses^31^. The survey was developed in English using B1 language to make items understandable for the general public^32^.

#### Survey evaluation (expert reviews and cognitive interviews)

The first survey draft's contents were systematically evaluated by four experts (medical entomologist, risk communication/questionnaire development specialist and native English speaking science communicators) using a rubric with the following criteria: clarity, wording, responses, appropriateness, balance, measure of constructs and survey aims^33,34^. Changes to the survey were made according to the experts’ scores and comments.

To test the survey within a target population, cognitive interviews with five United Kingdom (UK) residents were conducted through online videoconferencing, following existing guidelines^33^. The participants consented to participate in the study and to being recorded during the exercise. During the cognitive interviews, participants read items aloud and verbally narrated what they were thinking while selecting their answers. Researchers (AA and PdB) and participants flagged any items that seemed confusing or took long to answer. Flagged items, item wording and language and any other changes were discussed after completing the survey. Changes were made to the survey accordingly.

#### Translations

To test the survey in the other two target populations (residents of the Netherlands and Spain), the validated English survey (Fig. 1, Survey Version 3) was translated to Spanish and Dutch using forward–backward translation^34^. Items were translated to B1 levels of Spanish and Dutch, while keeping the same meaning of the items. Some items’ answer options (specifically for country of residence, province of residence, and travel destinations within Europe) had to be modified to match either the Dutch or Spanish target population. Once translated, both surveys were pilot tested in the new languages. Experts in virology and entomology in both countries completed the survey and provided feedback and suggestions to achieve functional equivalence. Additionally, after giving consent, a sample of 10–15 residents in each country (the Netherlands and Spain) completed the survey as a pre-test.

### Data collection

To assess the survey’s validity within our target populations, data was collected using heterogeneous, representative samples (based on age and sex) from the general populations of the United Kingdom, the Netherlands, and Spain aged 18 and over. Participants were recruited through two panels: Prolific Academic for UK residents and Bilendi for Dutch and Spanish residents^35,36^. Participants were directed from the panel environment to the survey in LimeSurvey. In LimeSurvey, participants were informed about the study aim, their right to withdraw from the study, and assured their data would be stored anonymously. By continuing with the survey, participants gave their informed consent. We included participants who met the following criteria: resident of the respective country (UK, the Netherlands or Spain) and aged ≥ 18 years.

The first version of the survey (Fig. 1, Survey Version 1) was completed by a sample of UK participants between October 4 and October 8, 2021 (UK Sample 1). Following the initial validity analysis, the survey was refined (Fig. 2, Survey Version 2) and distributed again to a second sample of UK citizens through Prolific from April 11 to April 14, 2022 (UK Sample 2). Participants in the second sample were asked if they had completed the survey in October and were excluded if they answered yes. To validate surveys in Spanish and Dutch, the translated surveys (Fig. 2, Survey Version 3) were distributed to participants in Spain and the Netherlands. Data was collected in three waves to ensure an even distribution of participants over the summer of 2022. Wave 1 was from July 19 to July 31, wave 2 was from August 1 to August 31 and wave 3 was from September 1 to September 30.

### Data analysis

#### Exclusion criteria and descriptive statistics

Before the data was analysed, exclusion criteria of participants were applied. Participants were excluded if (a) they incorrectly answered both control items (located in different parts of the survey), (b) they did not complete the survey in the appropriate time limit (2–25 min) or (c) they did not complete the full survey.

Descriptive statistics were performed for participant characteristics and item response frequencies for the HBM items using R (version 4.2.2, by RStudio version 2022.12.0 + 335 for Mac and 2022.07.1 + 554 for Windows; Posit Software, PBC, Boston, MA).

#### Confirmatory factor analysis and reliability testing

Survey validation was primarily completed using confirmatory factor analysis (CFA), performed using the lavaan package in R^37^. CFA tests how item response patterns relate to each other. By analysing response patterns, CFA can determine if items fit within the designed HBM constructs and if these items group together to measure each respective construct. To ensure the order of the HBM items in the survey would not influence the participants’ item response patterns, the items’ order was randomized within the online survey environment (LimeSurvey). Since multiple items measure the same construct and the item responses were categorical, we used a Weighted Least Squares with Mean and Variance adjustment estimator, referred to as WLS(MV) estimator^38,39^.

The goodness of fit of the HBM was assessed for each survey version (using participants’ responses) based on the following commonly used fit indices for CFA: chi-square test (*χ*^2^), root mean square error of approximation (RMSEA) ≤ 0.06 and comparative fit index (CFI) ≥ 0.95^40–42^. Since chi-square tests are sensitive to sample size, we use the ratio of the chi-square statistic to the degrees of freedom (*χ*^2^*/*df) to assess fitness, with a ratio of ≤ 2 indicating a good fit^43,44^. Furthermore, to aid item selection and to improve model fit, item factor loadings where assessed. Factor loadings show the variance explained by an item within the corresponding construct (also called a factor). Items with a low factor loading (< 0.3) with their corresponding constructs do not substantially contribute to explaining variance within that construct^45^. To improve the model fit parameters, items were removed from the model and then from the survey when: (a) the factor loading with the corresponding construct was less than 0.3 or (b) when modification indices indicated a better model fit if an item were to be removed^42,46^. To evaluate each construct’s and the survey’s reliability (consistency of the data), we used Cronbach’s alpha (α). Cronbach’s alpha is a measure of internal consistency and functions as an index of reliability^26^. Alpha values of 0.6–0.7 were considered acceptable, and values above 0.7 were considered good^47^.

#### HBM scores

Mean scores for each HBM construct were calculated using the 7-point Likert-scale, where “Strongly Agree” equated to 7 points and “Strongly Disagree” was worth 1 point. However, for Perceived Barriers, answers were inversed, so a higher Perceived Barriers mean score indicates there are no perceived barriers for prevention measure use and, thus, a higher intent to engage in preventive behaviour. All mean construct scores were summed into a final HBM score (with a score range of 6 to 42). A low HBM score reflects a low intent to show preventive behaviour, and a high score reveals a high intent to show preventive behaviour.

### Ethical statements

All methods were carried out in accordance with relevant guidelines and regulations for human subjects’ research, including European ethics requirements and best practices. Survey respondents were all at least 18 years old, they provided informed consent prior to participating, and no personal information was collected from them. The research protocol was submitted to the Medical Ethics Committee at Erasmus University Medical Center prior to implementation, and that Committee determined that the methods were not subject to the Dutch Medical Research Involving Human Subject’s Act and could therefore be carried out without further review (Ref. MEC-2021-0586 dated 21 September 2021).

## Results

### Survey evaluation (expert reviews and cognitive interviews)

After the HBM was selected as the survey model, items were generated, and a first survey draft was created containing 64 items (Fig. 1). Survey evaluation through experts’ rubric evaluations and cognitive interviews resulted in removing 15 items. Suggestions were mostly directed at avoiding negative wording, specifying items to ensure correct item interpretation and clarifying by adding pictures. Modifications resulted in Survey Version 1 with a total of 57 items, including 25 HBM items (Figs. 1 and 2, Survey Version 1).

#### Participant characteristics

After applying the exclusion criteria, the final number of participants were 513 and 338 for the United Kingdom (first and second sample) and 438 and 475 for the Netherlands and Spain, respectively. Table 1 shows the demographic characteristics of the included participants for each data collection sample and the national median age^48^. The participants in the Netherlands sample had the same median age as that of the Dutch population aged 18 and over. The gender distribution of the Netherlands sample matches the national gender distribution for this age range (Male 49%, Female 51%)^48^. The Spanish national median age for residents aged 18 and over is one year higher than the median age in the Spain sample. The gender distribution in the Spain sample shows slightly more male participants compared to the national distribution for this age range (Male 48.3%, Female 51.7%). The UK national median age for residents aged 18 and over is three years higher than the median age of each of the UK samples. The gender distribution in the UK sample shows a slightly lower proportion of male participants compared to the national distribution for this age range^48^.

**Table 1:** Characteristics of participants from countries of survey distribution.

Characteristics	UK1	UK2	NL	ES
	N = 513	N = 338	N = 438	N = 475
Age				
National median^a^	48	48	49	50
Median	45	45	49	49
(Min–max)	(18–84)	(18–84)	(18–99)	(18–89)
IQR	28	27	35.5	26
Gender				
National percent male	51.40%	51.40%	51.10%	51.70%
Male	246 (48%)	164 (49%)	213 (49%)	231 (49%)
Female	261 (51%)	171 (51%)	225 (51%)	242 (51%)
Other	4 (1%)	3 (1%)	1 (0.2%)	2 (0.4%)

*UK1* United Kingdom Sample 1, *UK2* United Kingdom Sample 2, *NL* Netherlands, *ES* Spain.

^a^National median age and percent male for residents 18 years and older calculated from Eurostat population data from 2022 (Netherlands and Spain) and 2019 (UK).

### Confirmatory factor analysis and reliability testing

The validity of Survey Version 1 was assessed by performing CFA to assess if the designed items fit the respective HBM constructs with the item responses collected from the UK Sample 1 (n = 513). Model A (Table 2) showed that item responses did not fit the constructs, as factor loadings were too low for several items. Item removal to optimize the model fit by assessing multiple models resulted in model F (Table 2), which showed acceptable fit indices (*χ*^2^*/*df = 2.18, RMSEA = 0.048 and CFI = 0.931). Additionally, Model F shows acceptable reliability (α = 0.69). Additional items were created and some rephrased based on factor loadings (Supplementary Table 1) and item response frequencies (Supplementary Fig. 1). The modified survey resulted in 22 HBM items (Supplementary Material, Full Surveys).

**Table 2:** Confirmatory factor analysis and reliability test results for the item responses from samples in the United Kingdom, the Netherlands, and Spain.

Sample (survey version)	Model (number of items)	*χ*^2^	df	*χ*^2^*/df*	RMSEA	RMSEA 95%	CFI	α
UK Sample 1 (survey version 1)	Model A (25)	1173.02	265	4.43	0.08	0.08–0.09	0.72	0.73
	Model C (20)	481.57	160	3.01	0.06	0.06–0.07	0.85	0.71
	Model D (19)	401.09	125	3.21	0.07	0.06–0.07	0.86	0.72
	Model E (18)	282.95	109	2.6	0.06	0.05–0.07	0.91	0.71
	Model F (17)	204.99	94	2.18	0.05	0.04–0.07	0.93	0.69
UK Sample 2 (survey version 2)	Model G (22)	368.32	199	1.85	0.05	0.04–0.06	0.93	0.74
	Model H (21)	318.91	179	1.78	0.05	0.04–0.06	0.94	0.75
	Model I (21)	257.39	174	1.48	0.04	0.03–0.05	0.96	0.75
	Model J (20)	230.75	155	1.49	0.04	0.03–0.05	0.97	0.76
UK Sample 2 (survey version 3)	Model K^UK^ (19)	149.05*	137	1.09	0.02	0.00–0.03	0.99	0.73
NL (survey version 3)	Model K^NL^ (19)	239.51	137	1.75	0.04	0.03–0.05	0.95	0.76
ES (survey version 3)	Model K^ES^ (19)	165.61	137	1.21	0.02	0.00–0.04	0.99	0.79

*χ*^2^ Chi-square value. *rmsea* root mean square error of approximation.

*CFI* Comparative Fit Index, *α* Cronbach’s alpha, *UK* United Kingdom, *NL* Netherlands, *ES* Spain, *EN* English.

*Indicates a nonsignificant *P* value above 0.05. Values were considered good when* χ*^2^/df ≤ 2, RMSEA ≤ 0.06 and CFI ≥ 0.95. Cronbach Alpha values of 0.6–0.7 were considered acceptable, and values above 0.7 were considered good.

The validity of Survey Version 2 was assessed by performing CFA with the item responses from UK population sample 2 (n = 338) (Table 2, Model G). Model G revealed the data did not fit since the thresholds were not met. After closer assessment, Model G showed that SEVrecover did not contribute to Perceived Severity (factor loading = − 0.059) (Table 3) and was removed, resulting in Model H (Table 2). Model H showed improved fit indices, but the data did not fit the five-construct structure (Table 2). Factor loading revealed an underlying latent trait within the combined Perceived Barriers and Benefits construct, leading to a new six-construct model structure in Model I (Table 2). This modification, together with the removal of two additional items (BBcloth and SEpmuse) due to low factor loadings, resulted in the new six-construct, 19-item Model K^UK^. Model K^UK^ showed good model fit (*χ*^2^*/*df = 1.09, RMSEA = 0.016 and CFI = 0.993) (Table 2), factor loadings (Table 3) and overall scale reliability (α = 0.73). All constructs of the final Model K^UK^ showed acceptable and good reliability, except for Perceived Benefits (α = 0.46) (Table 4). Since all other measurement properties, including the factor loadings of the Perceived Benefits, indicated a good fit, Model K^UK^ was accepted. Survey Version 3 was comprised of these 19 HBM items from Model K^UK^, which were translated to Dutch (Model K^NL^) and Spanish (Model K^ES)^ (Fig. 2, Survey Version 3).

**Table 3:** Factor loadings results after distribution of Survey Version 2 in the United Kingdom (Sample 2).

Item code	Item	Factor loadings (survey version 2)				
		Model G	Model H	Model I	Model J	Model K
SUSbite	The likelihood to be bitten by a mosquito in my country of residence is high	0.685	0.685	0.687	0.684	0.687
SUSres	I am at risk of getting infected with a mosquito-borne virus in my country of residence	0.618	0.618	0.616	0.617	0.630
SUSmbv	I am worried about getting sick from a mosquito-borne virus in my country of residence	0.731	0.731	0.727	0.731	0.721
SUSmosq	I live in a neighbourhood where mosquitoes are highly present	0.731	0.731	0.735	0.732	0.726
SEVprobs	Getting sick with a mosquito-borne virus may result in hospitalisation	0.714	0.717	0.710	0.712	0.712
SEVdeadly	People can die from a mosquito-borne virus infection	0.758	0.766	0.768	0.767	0.768
SEVqual	Becoming sick from a mosquito-borne virus can reduce your ability to do daily tasks	0.649	0.644	0.648	0.647	0.647
SEVrecover	The chance of recovering from a mosquito-borne virus is high	− 0.059	NA	NA	NA	NA
BBbites	Using skin repellents (such as DEET) prevents mosquito bites	0.429	0.427	0.519	0.515	0.535
BBsafe	Skin repellents (such as DEET) are safe to use	0.331	0.328	0.370	0.372	0.383
BBprev	If I use preventive measures, I will avoid getting bitten by mosquitoes	0.431	0.428	0.546	0.548	0.520
BBcloth	In hot weather, wearing long-sleeved and long trousers as a prevention measure against mosquito bites is uncomfortable	0.099	0.102	0.177	NA	NA
BBannoy	Using mosquito preventive measures is more annoying than mosquitoes themselves	0.347	0.351	0.559	0.556	0.566
BBtime	Applying prevention measures takes too much time	0.505	0.509	0.836	0.792	0.779
CUEnotif	Getting news alerts about mosquito-borne virus cases in my area would remind me to use preventive measures	0.410	0.410	0.410	0.411	0.429
CUEout	During the summer, going outside reminds me to use prevention measures against mosquitoes	0.789	0.790	0.787	0.787	0.758
CUEmosq	Mosquitoes in and around my house at night remind me to use prevention measures against mosquitoes	0.650	0.650	0.652	0.652	0.665
SEbreedid	I am confident I can identify mosquito breeding sites	0.515	0.514	0.517	0.517	0.634
SEbreedrem	I am confident I can remove mosquito breeding sites in and around my house during mosquito season (March to September)	0.491	0.491	0.489	0.489	0.583
SEpmuse	I remember to apply preventive measures against mosquitoes during mosquito season (March to September)	0.746	0.747	0.741	0.742	NA
SEinfo	I know where to find information about prevention measures against mosquito bites	0.439	0.439	0.441	0.441	0.490
SEbest	I know which prevention measures are best to use against mosquito bites	0.677	0.676	0.683	0.681	0.758

Items with factor loadings < 0.3 were removed from the survey. Models G and H have five HBM constructs. Models I, J and K have six HBM constructs since Perceived Barriers and Perceived Benefits have been separated into two constructs.

**Table 4:** Factor loadings and construct reliability results for the final Health Belief Model items (Model K) for the United Kingdom, the Netherlands and Spain.

Construct	Item code	Model K^EN^		Model K^NL^		Model K^ES^	
		Factor loadings	α	Factor loadings	α	Factor loadings	α
Perceived Susceptibility	SUSmosq	0.726	0.79	0.492	0.57	0.615	0.73
	SUSbite	0.687		0.438		0.558	
	SUSmbv	0.721		0.571		0.716	
	SUSres	0.630		0.436		0.656	
Perceived Severity	SEVprobs	0.712	0.75	0.871	0.81	0.805	0.77
	SEVdeadly	0.768		0.663		0.624	
	SEVqual	0.647		0.772		0.757	
Perceived Benefits	BBbites	0.535	0.46	0.693	0.66	0.760	0.68
	BBsafe	0.383		0.491		0.574	
	BBprev	0.520		0.658		0.602	
Perceived Barriers	BBannoy	0.566	0.61	0.452	0.63	0.779	0.63
	BBtime	0.779		1.009		0.558	
Self-Efficacy	SEbest	0.758	0.71	0.796	0.75	0.747	0.78
	SEinfo	0.490		0.622		0.603	
	SEbreedid	0.634		0.636		0.676	
	SEbreedrem	0.583		0.585		0.721	
Cues to Action	CUEout	0.758	0.64	0.765	0.69	0.632	0.66
	CUEmosq	0.665		0.675		0.709	
	CUEnotif	0.429		0.504		0.552	

*EN* United Kingdom, *NL* Netherlands, *ES* Spain, *α* Cronbach’s alpha coefficient.

The validity of Dutch Survey Version 3 was assessed by performing CFA with the item responses from the Dutch sample (n = 438). CFA confirmed an acceptable fit of Model K^NL^ (Table 2), and all factor loadings were above 0.3 (Table 4). Constructs showed acceptable or good reliability except for Perceived Susceptibility (α = 0.57) (Table 4). The overall reliability of the HBM survey was also good (α = 0.76).

By performing CFA with the item responses from the Spanish sample (n = 475), the validity of the Spanish Survey Version 3 was also assessed. CFA indicated a good fit of Model K^ES^ (Table 2), and all factor loadings were above 0.3 (Table 4). Each construct showed acceptable or good reliability (Table 4). Reliability of the full Spanish HBM scale was also good with a Cronbach’s alpha coefficient of 0.79.

#### Final survey

The final Survey Version 3 has 57 items, which includes the 19 validated HBM items, and is available in three languages (English, Dutch and Spanish) (Supplementary Material, Full Surveys). Table 5 shows all the HBM items in the final survey version. The additional items (not the validated HBM items) are directed at identifying potential characteristics that might influence the behaviour as measured by the HBM items and include: Predictor items (including direct surroundings and housing, travelling, mosquito nuisance and information sources) (n = 26), knowledge items (mosquito biting times, breeding sites, MBV, and MBV transmission routes) (n = 4), prevention measure use (which prevention measures used and reasons for using or not using prevention measures) (n = 3), perceived responsibility (n = 3) and the control items (n = 2).

**Table 5:** Survey Version 3, the Health Belief Model items in English, Dutch and Spanish.

Construct	Item code	English	Dutch	Spanish
Perceived Susceptibility	SUSmosq	I live in a neighborhood where mosquitoes are highly present	Ik woon in een buurt waar veel muggen zijn	Vivo en un barrio donde hay muchos mosquitos
	SUSbite	The likelihood of being bitten by a mosquito in my country of residence is high	De kans om in het land waar ik woon gebeten te worden door een mug is groot	La probabilidad de ser picado por un mosquito en mi país de residencia es alta
	SUSmbv	I am worried about getting sick from a mosquito-borne virus in my country of residence	Ik maak me zorgen dat ik in het land waar ik woon ziek word van een door muggen overdraagbaar virus	Me preocupa contraer una enfermedad causada por un virus transmitido por mosquito en mi país de residencia
	SUSres	I am at risk of getting infected with a mosquito-borne virus in my country of residence	Ik loop het risico besmet te raken met een door muggen overdraagbaar virus in het land waar ik woon	Estoy en riesgo de infectarme con un virus transmitido por mosquitos en mi país de residencia
Perceived Severity	SEVprobs	Getting sick with a mosquito-borne virus may result in hospitalisation	Ziek worden van een door muggen overdraagbaar virus kan een ziekenhuisopname als gevolg hebben	Enfermarse con un virus transmitido por mosquitos puede resultar en la hospitalización
	SEVdeadly	People can die from a mosquito-borne virus infection	Mensen kunnen overlijden aan een besmetting met een door muggen overdraagbaar virus	Las personas pueden morir a causa de una infección por un virus transmitido por mosquitos
	SEVqual	Getting sick from a mosquito-borne virus can reduce your ability to do daily tasks	Ziek worden van een door muggen overdraagbaar virus kan je vermogen om dagelijkse taken uit te voeren verminderen	Al enfermarme a causa de un virus transmitido por mosquito, mi capacidad para realizar tareas diarias puede verse reducida
Perceived Benefits	BBbites	Applying repellents on the skin (such as DEET) prevents mosquito bites	Het gebruiken van insectenspray (zoals DEET) op de huid voorkomt muggenbeten	El uso de repelentes para la piel (como DEET) previene las picaduras de mosquitos
	BBsafe	Repellents applied on the skin (such as DEET) are safe to use	Insectenspray voor op de huid (zoals DEET) is veilig om te gebruiken	Los repelentes para la piel (como el DEET) son seguros de usar
	BBprev	If I use prevention measures, I will avoid getting bitten by mosquitoes	Als ik voorzorgsmaatregelen neem voorkom ik dat ik door muggen wordt gebeten	Si utilizo medidas preventivas, evitaré que me piquen los mosquitos
Perceived Barriers	BBannoy	Using prevention measures against mosquitoes is more annoying than mosquitoes themselves	Voorzorgsmaatregelen nemen tegen muggen is vervelender dan de muggen zelf	Usar medidas de prevención contra los mosquitos es más irritantes que los mosquitos en sí
	BBtime	Applying prevention measures takes too much time	Voorzorgsmaatregelen nemen tegen muggen kost te veel tijd	Aplicar las medidas de prevención contra los mosquitos lleva demasiado tiempo
Self-Efficacy	SEbest	I know which prevention measures are best to use against mosquito bites	Ik weet welke voorzorgsmaatregelen ik het beste kan nemen tegen muggenbeten	Sé que medidas de prevención son mejores contra las picaduras de mosquito
	SEinfo	I know where to find information about prevention measures against mosquito bites	Ik weet waar ik informatie kan vinden over voorzorgsmaatregelen tegen muggenbeten	Sé dónde encontrar información sobre medidas de prevención contra las picaduras de mosquito
	SEbreedid	I am confident I can identify mosquito breeding sites	Ik ben er zeker van dat ik muggenbroedplaatsen kan identificeren	Confío en que puedo identificar criaderos de mosquitos
	SEbreedrem	I am confident I can remove mosquito breeding sites in and around my house during mosquito season (March to September)	Ik ben er zeker van dat ik muggen broedplaatsen in- en om mijn huis kan verwijderen tijdens het muggenseizoen (maart tot en met september)	Confío en que puedo eliminar criaderos de mosquitos dentro y alrededor de mi casa durante la temporada de mosquitos (de marzo a septiembre)
Cues to Action	CUEout	During the summer, going outside (hiking in nature, camping, picnics, gardening) reminds me to use prevention measures against mosquitoes	Naar buiten gaan in de zomer (zoals wandelingen in de natuur, kamperen, picknicks, tuinieren) herinnert mij eraan om voorzorgsmaatregelen te nemen tegen muggen	Durante el verano, estar en espacios exteriores (senderismo en la naturaleza, de acampada, de pícnic, al jardín) me recuerda que tengo que usar medidas preventivas contra mosquitos
	CUEmosq	Mosquitoes in and around my house at night remind me to use prevention measures against mosquitoes	Muggen in- en om mijn huis ‘s nachts herinneren me eraan om voorzorgsmaatregelen te nemen tegen muggen	Los mosquitos dentro y alrededor de mi casa por la noche me recuerdan que debo usar medidas de prevención contra los mosquitos
	CUEnotif	Getting news alerts about mosquito-borne virus cases in my area would remind me to use prevention measures	Als ik nieuwsberichten ontvang over ziektegevallen van een door muggen overdraagbaar virus in mijn regio, zou ik eraan herinnerd worden om voorzorgsmaatregelen te nemen	Recibir alertas de noticias sobre casos de virus transmitidos por mosquitos en mi área me recordaría usar medidas de prevención

#### HBM scores

Using the responses to the 19 validated HBM items, construct mean scores and HBM sum scores were calculated for each participant per country (Table 6).

**Table 6:** Overview of scores per construct and total HBM score for the United Kingdom (Sample 2), the Netherlands and Spain.

Construct	United Kingdom sample 2			Netherlands			Spain		
	n = 336			n = 438			n = 475		
	Mean	Median	Q1–Q3	Mean	Median	Q1–Q3	Mean	Median	Q1–Q3
Perceived Susceptibility	2.41	2.25	1.50–3.25	4.04	4	3.50–4.50	4.22	4.25	3.50–5.00
Perceived Severity	5.99	6	5.67–6.67	5.06	5	4.00–6.00	5.4	5.67	4.67–6.00
Perceived Benefits	4.88	5	4.33–5.33	4.97	5	4.33–5.67	5.32	5.33	4.67–6.00
Perceived Barriers	5.1	5	4.50–6.00	4.57	4.5	4.00–5.50	4.38	4.5	3.50–5.50
Self-Efficacy	4.13	4.25	3.25–5.00	4.57	4.5	4.00–5.25	4.4	4.5	3.75–5.25
Cues to Action	4.34	4.33	3.33–5.33	4.98	5	4.33–6.00	5.39	5.33	4.67–6.00
Total HBM	26.8	26.9	24.58–29.17	28.2	28.2	25.75–30.58	29.1	29.1	26.75–31.50

The construct score range is 1–7. The total HBM score range is 6–42.

*Q1* first quartile, *Q3* third quartile, *HBM* Health Belief Model.

## Discussion

This study describes the development and validation of an HBM-based survey to assess perceptions of mosquito bites and MBVs in European regions. We developed the MosquitoWise survey, the first validated Europe-wide applicable tool to measure these constructs among Europe’s residents. With the (expected) expanding range of mosquito-borne diseases, this survey can aid in understanding and creating data-driven decisions to alter populations’ behaviours to prevent mosquito-borne disease transmission.

The development of the MosquitoWise survey makes several contributions to the currently available European surveys. Previously developed surveys have tended to be related to a specific mosquito species or MBV, rather than a broader scope of understanding behaviour towards mosquitoes and MBVs. Additionally, most surveys used in European studies are not validated, are Knowledge, Attitudes and Practices (KAP) surveys or are tailored to a certain population, rather than the general public^17,18,21,49,50^. We identified one Italian validated survey, created specifically to measure knowledge, attitude and behaviours towards Zika in the general population. While the necessary steps were taken to validate this survey, the survey was validated using responses from medical doctors, who were not the intended target population^51^. Although this gives some insight into the survey’s internal consistency, the survey might perform differently in the intended population. Since our survey’s target population is the public, we ensured that validity analysis was based on responses from the general population in the three targeted countries. This helps make the MosquitoWise survey particularly useful for research in Europe’s shifting MBV landscape.

This study has shown that the analysis of content and constructs have clearly improved the survey’s performance as measured by the confirmatory factor analyses and internal consistency reliability testing. This is exemplified by the restructuring of the construct Perceived Barriers and Benefits into two separate constructs. While the first and second survey versions followed the combined structure, factor loadings for Survey Version 2 indicated that the survey would perform better with Perceived Barriers and Perceived Benefits as separate constructs. This change improved the performance of the survey, as assessed by the fit indices of the confirmatory factor analyses performed for the different countries, resulting in the final version of this survey (Fig. 2, Survey Version 3). While the reliability of most constructs was between 0.6 and 0.81, Cronbach’s alpha for Perceived Susceptibility in the Dutch survey was lower but very close to 0.6. The Cronbach's alpha for Perceived Benefits is below 0.5 for the English version of the survey. The number of items in a construct influences the Cronbach alpha, so constructs with a lower number of items more often show lower values^47^. Increasing the number of items could, therefore, be a solution. However, the survey length is also an important consideration together with the other measurement properties of the items. Further research in the United Kingdom could help improve the performance of the construct in this country. Since the overall reliabilities for the survey scales for the United Kingdom, the Netherlands and Spain are within a good range, we accept the reliability of the surveys.

Some limitations of this study need to be acknowledged. We aimed to assess the surveys validity and reliability using a representative population sample. The panels used in this survey were chosen as effective ways to reach participants, but using survey panels has several limitations. First, representativeness can never fully be achieved within a panel population. Although the median ages and gender distribution of our sampled populations and the national median ages and gender distributions are closely related, other non-corrected population characteristics might show under or overrepresentation^52^. Furthermore, panel members are usually people who already have an interest in completing surveys or join the panel for incentives. Thus, their responses could be influenced by either of these factors, a phenomenon known as panel conditioning^53^. We tried to account for this phenomenon by selecting panels created using probabilistic recruitment, meaning the panel provider randomly invites people from the general population to become a panellist to reduce the effects of panel conditioning^54^.

Lastly, our study may have been subject to recall bias, as participants were asked to report their perceptions and behaviour during mosquito season. We attempted to minimize recall bias by distributing the survey during peak mosquito season months (July and August). However, this was not possible for data collection in the UK. Data collection for Sample 1 took place in April (beginning of mosquito season) and Sample 2 in October (end of mosquito season)^55,56^. Nevertheless, we expect potential recall bias effects to be small, since the period (mosquito season) was clearly specified in each item where this was relevant and mosquito seasonality greatly differs by region^57^.

Despite these limitations, having a validated survey establishes a standard for measurement by ensuring that items are clear, well-understood and measure the intended outcome in the target population. Already having a standard in place reduces time needed to create a survey, enhancing efficiency and consistency. This survey has undergone expert reviewing, pre-testing and has been tested in the target population four times (Fig. 1), diminishing the likelihood of measurement errors and improving data accuracy and reliability of responses.

After careful selection of items and a comprehensive validation process, the MosquitoWise survey is ready to use in Europe. Aside from the survey’s 19 core HBM items, which are validated and cannot be changed without revalidating the tool, the knowledge and demographic questions can be removed, added or adjusted to better suit researchers’ aims and local situations. Thus, the tool is adaptable and versatile by adding complementary potential background variables that may influence or predict the measured behaviour or knowledge based on specific goals. Not only can this survey be adapted by adding demographic and knowledge questions, but it can also be focused on specific mosquito species or MBVs. While this modification would require revalidating the adjusted survey, the items’ phrasal structure allows for this easy change, saving time in survey development. For example, the word “mosquito” in the item ‘*The likelihood of being bitten by a mosquito in my country of residence is high*’ can easily be replaced with '*tiger mosquito*', or any other species, keeping the exact same structure of the item while focusing on a specific mosquito species. Similarly, ‘*Getting sick with a mosquito-borne virus may result in hospitalisation*’ can be altered to a specific MBV like Zika or dengue virus.

Furthermore, since this tool is validated and is available in English, Dutch and Spanish, comparing survey data from countries with a different situational background is possible and can provide insight into health behaviours. To ensure that multiple countries’ data can be compared, we suggest sampling populations at similar time points (especially during mosquito season) and assessing measurement invariance between countries^58^. These results can be used to optimize preparedness policies and communication to the public. The use of this tool at multiple time points can provide insight on behaviour change over time, by measuring the effects and evaluation of communication campaigns, for instance. This can be especially useful considering situational changes resulting in increasing MBV exposure and risk. Combining the survey with entomological or serological research provides further options to quantify effects of residents’ knowledge and preventative behaviours on matters such as larvae presence in backyards or MBV exposure in populations^20,21,59,60^.

With environments changing and becoming more suitable for mosquito expansion, understanding people’s perceptions is crucial to prevent invasive mosquito species establishment and the potential for disease transmission. Thus, recognizing this relationship early can serve as an effective method for successful behavioural interventions in outbreak prevention and management. Our MosquitoWise survey fills a clear gap in knowledge, not only on a national scale, but on a continental one.

### Supplementary Information

Supplementary Information.

Supplementary Figure 1.

Supplementary Table 1.

## Data Availability

The datasets generated and analysed during the current study are available from the corresponding author on reasonable request.
