# Supplementary material for: Development and validation of the MosquitoWise survey to assess perceptions towards mosquitoes and mosquito-borne viruses in Europe
Source: Sci Rep. 2024 Jan 20;14:1777. doi: 10.1038/s41598-024-52219-9 (PMC10799950; doi:10.1038/s41598-024-52219-9)
Supplement: Supplementary file 1 — Supplementary Information. [file 41598_2024_52219_MOESM1_ESM.pdf]

**Full Surveys.** Survey questions, answer choices and structure.
Answer types:

- ○ Multiple choice, one answer
- ❑ Multiple choice, multiple answers possible

## Final Survey Version - English

**First page of Survey**

**Survey Title**

**Background information on the purpose of the research and the use of the survey responses.**

***The purpose of this survey is to better understand your knowledge and perceptions of mosquitoes and mosquito-borne viruses in Europe. The aim of this survey is also to understand what current preventive measures you use to avoid being bitten by mosquitoes.***

***This survey is intended for people 18 years and older who live in the United Kingdom. This survey should take no more than 15 minutes.***

***Your participation is completely voluntary, and all answers will remain anonymous. By continuing, you agree to participate in this survey and give us permission to save your anonymous answers for analysis.***

***Your answers will help us greatly. Thank you so much for your time!***

**Section 1. Your Surroundings and Experiences with Mosquitoes**

**There are many different mosquito species. Some mosquito species bite people, in this survey we will focus on mosquitoes that bite. Throughout the survey, we will just call them mosquitoes. The questions in this section are about your experiences with mosquitoes in and around your house.**

| Question code | Question | Answer Choices |
|---|---|---|
| EXage | Are you 18 years old or older? | ○ Yes > Conditional. Survey ends due to exclusion criteria.<br>○ No |
| DEMout | Does your residence include outdoor space you use (like a garden, balcony or roof terrace)? | ❑ Yes<br>❑ No |
| DEMactive | I experience mosquito nuisance (annoyance) during mosquito season (March to September) during the following activities:<br><br>(Select all that apply) | ❑ Spending time outside around my house (e.g. garden, balcony) > Conditional. If selected, go to DEMactive1<br>❑ Playing sports/exercise outside (e.g. football, running) > Conditional. If selected, go to DEMactive2<br>❑ Being outside for leisure activities (e.g. picnics, camping, hiking) > Conditional. If selected, go to DEMactive3<br>❑ Working > Conditional. If selected, go to DEMactive4 |

| | | |
|---|---|---|
| | | ❑ Sleeping > Conditional. If selected, go to DEMactive5<br>❑ Travelling for education or work purposes (work commute) > Conditional. If selected, go to DEMactive6<br>❑ Other<br>❑ I do not experience any mosquito nuisance during the above listed activities. |
| DEMactive1 | (Conditional) How frequently do mosquitoes annoy you when you spend time outside around your house (e.g. garden, balcony)? | ○ Very frequently<br>○ Frequently<br>○ Occasionally<br>○ Rarely |
| DEMactive2 | (Conditional) How frequently do mosquitoes annoy you when you play sports/exercise outside (e.g. football, running)? | ○ Very frequently<br>○ Frequently<br>○ Occasionally<br>○ Rarely |
| DEMactive3 | (Conditional) How frequently do mosquitoes annoy you when you are outside for leisure activities (e.g. picnics, camping, hiking) | ○ Very frequently<br>○ Frequently<br>○ Occasionally<br>○ Rarely |
| DEMactive4 | (Conditional) How frequently do mosquitoes annoy you when you are working? | ○ Very frequently<br>○ Frequently<br>○ Occasionally<br>○ Rarely |
| DEMactive5 | (Conditional) How frequently do mosquitoes annoy you when you are sleeping? | ○ Very frequently<br>○ Frequently<br>○ Occasionally<br>○ Rarely |
| DEMactive6 | (Conditional) How frequently do mosquitoes annoy you when you are travelling for education or work purposes (work commute)? | ○ Very frequently<br>○ Frequently<br>○ Occasionally<br>○ Rarely |
| **Section 2. Your Understanding of Mosquitoes and Mosquito-Borne Viruses**<br><br>**This section contains statements about mosquito biology, their breeding sites and mosquito-borne viruses.**<br><br>Questions in this section are randomized. | | |
| **Question code** | **Question** | **Answer Choices** |
| KNbite | Mosquitoes only bite people during the day. | ○ Yes<br>○ No<br>○ I do not know. |
| KNbreed | In gardens, mosquitoes can lay eggs in:<br><br>(Select all that apply) | ❑ Standing water in a bucket<br>❑ B. Leftover food on the garden table<br>❑ C. Standing water in gutters<br>❑ None of the above<br>❑ I do not know. |

| | | |
|---|---|---|
| | 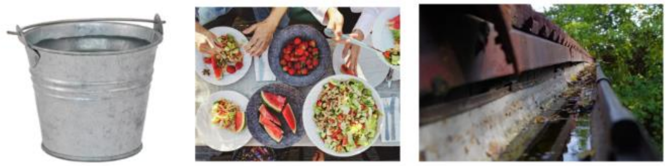<br>A. Still standing water in bucket   B. Leftover food on garden table   C. Still standing water in gutters | |
| KNvirus | Mosquitoes are the main spreaders of the following viruses:<br><br>(Select all that apply) | ❑ West Nile virus<br>❑ Chikungunya virus<br>❑ Zika virus<br>❑ Influenza (Flu)<br>❑ Dengue virus<br>❑ Human imunodeficiency virus (HIV)<br>❑ Measles virus<br>❑ None of the above<br>❑ I do not know. |
| KNroute | A person can possibly get a mosquito-borne virus if:<br><br>(Select all that apply) | ❑ They are bitten by an infected mosquito.<br>❑ They touch an infected person.<br>❑ They touch an infected animal.<br>❑ They get infected through the air<br>❑ They have sexual contact with an infected person.<br>❑ None of the above<br>❑ I do not know. |

**Section 3: Prevention Measures against Mosquitoes**

**For this section, please answer the questions in relation to your experiences in and around your house. All questions in this section are related to prevention measures you use against mosquitoes.**

**After answering the questions about potential breeding sites, we would like to give you some more information about mosquito breeding sites.**

**Mosquitoes can lay eggs in still water such as in a bucket or watering can in your garden, still water in clogged gutters or a rain barrel that is left uncovered.**

**Mosquito eggs grow to be adult mosquitoes in 14 days and need water for that. If you remove standing water every week, you can prevent mosquito eggs from growing to adult mosquitoes. This can reduce mosquito populations around your house.**

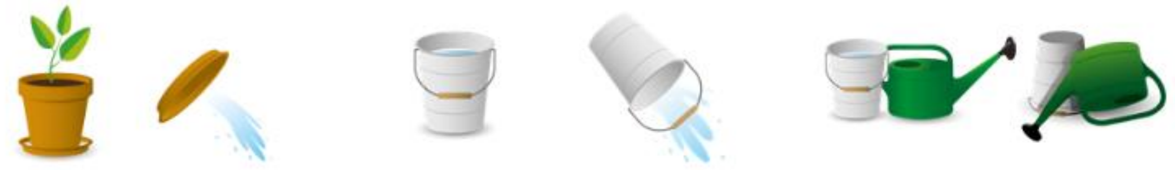

Source image: NVWA muggen

| Question code | Question | Answer Choices |
|---|---|---|
| PMuse | Check all the prevention measures that you use against mosquitoes:<br>(Select all that apply) | ❑ Wearing long sleeved shirts and trousers > Conditional. If selected, go to PMreasonY<br>❑ Using mosquito repellent on my body (such as DEET) > Conditional. If selected, go to PMreasonY<br>❑ Using an electric zapper > Conditional. If selected, go to PMreasonY<br>❑ Using an electric fan or air conditioning > Conditional. If selected, go to PMreasonY<br>❑ Using an outlet plug-in mosquito repellent > Conditional. If selected, go to PMreasonY<br>❑ Placing screens over doors and/or windows > Conditional. If selected, go to PMreasonY<br>❑ Sleeping with bedroom windows closed > Conditional. If selected, go to PMreasonY<br>❑ Sleeping under mosquito bed netting > Conditional. If selected, go to PMreasonY |

| | | |
|---|---|---|
| | | ❑ Using natural methods: garlic, herbs, plants > Conditional. If selected, go to PMreasonY<br>❑ Removing still water from places in and around my house > Conditional. If selected, go to PMreasonY<br>❑ Other > Conditional. If selected, go to PMreasonY<br>❑ None > Conditional. If selected, go to PMreasonN |
| PMreasonN | (Conditional)<br>For which reason(s) do you not use prevention measures against mosquitoes?<br>(Select all that apply) | ❑ There are no mosquitoes where I live.<br>❑ Prevention measures are annoying.<br>❑ Prevention measures are time-consuming.<br>❑ Mosquitoes do not bite me.<br>❑ Prevention measures are too expensive.<br>❑ Other |
| PMreasonY | (Conditional)<br>For which reason(s) do you use these prevention measures against mosquitoes?<br>(Select all that apply) | ❑ To reduce being bitten by mosquitoes<br>❑ To reduce number of mosquitoes in and around my house<br>❑ To reduce chance of getting a mosquito-borne virus<br>❑ Other |
| RESpp | Who do you think should be responsible for informing the public about prevention measures against mosquitoes?<br>(Select all that apply) | ❑ Government including health ministries<br>❑ Health professionals (such as nurses and doctors)<br>❑ Citizens themselves |
| RESpbreed | I think I am mainly responsible for removing mosquito breeding sites in and around my house. | ○ Strongly agree<br>○ Agree<br>○ Somewhat agree<br>○ Neutral<br>○ Somewhat disagree<br>○ Disagree<br>○ Strongly disagree |

| | | |
|---|---|---|
| RESgovbreed | I think the government* is mainly responsible for removing mosquito breeding sites in my neighbourhood.<br><br>* (i.e. local municipalities, health ministries and their related institutes) | ○ Strongly agree<br>○ Agree<br>○ Somewhat agree<br>○ Neutral<br>○ Somewhat disagree<br>○ Disagree<br>○ Strongly disagree |

**Section 4. Your Opinion about Prevention Measures**

**In this section, we ask questions on your opinion about using certain prevention (against mosquito bites).**

<u>Questions within this section are randomized</u>

| Question code | Question | Answer Choices |
|---|---|---|
| BENbites | Applying repellents on the skin (such as DEET) prevents mosquito bites.<br>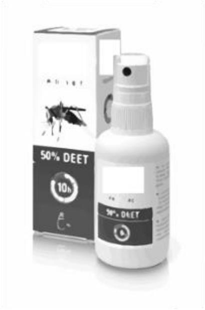<br> | ○ Strongly agree<br>○ Agree<br>○ Somewhat agree<br>○ Neutral<br>○ Somewhat disagree<br>○ Disagree<br>○ Strongly disagree |
| BENsafe | Repellents applied on the skin (such as DEET) are safe to use.<br>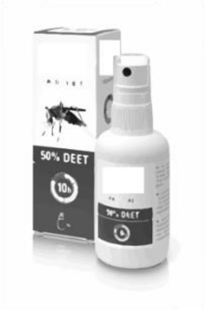<br> | ○ Strongly agree<br>○ Agree<br>○ Somewhat agree<br>○ Neutral<br>○ Somewhat disagree<br>○ Disagree<br>○ Strongly disagree |
| BENprev | If I use prevention measures, I will avoid getting bitten by mosquitoes. | ○ Strongly agree<br>○ Agree<br>○ Somewhat agree<br>○ Neutral<br>○ Somewhat disagree<br>○ Disagree<br>○ Strongly disagree |

| | | |
|---|---|---|
| BARannoy | Using prevention measures against mosquitoes is more annoying than mosquitoes themselves. | ○ Strongly agree<br>○ Agree<br>○ Somewhat agree<br>○ Neutral<br>○ Somewhat disagree<br>○ Disagree<br>○ Strongly disagree |
| BARtime | Applying prevention measures takes too much time. | ○ Strongly agree<br>○ Agree<br>○ Somewhat agree<br>○ Neutral<br>○ Somewhat disagree<br>○ Disagree<br>○ Strongly disagree |
| **Section 5. Mosquitoes and Mosquito-Borne Viruses**<br><br>**This section of the survey is about your experiences in and around your house and in your country of residence. We aim to understand how mosquito nuisance (annoyance), prevention measures and mosquito-borne viruses affect these experiences and your views on mosquitoes and mosquito-borne viruses.**<br><br><u>Questions within this section are randomized.</u> | | |
| **Question code** | **Question** | **Answer Choices** |
| SUSmosq | I live in a neighbourhood where mosquitoes are highly present. | ○ Strongly agree<br>○ Agree<br>○ Somewhat agree<br>○ Neutral<br>○ Somewhat disagree<br>○ Disagree<br>○ Strongly disagree |
| SUSbite | The likelihood of being bitten by a mosquito in my country of residence is high. | ○ Strongly agree<br>○ Agree<br>○ Somewhat agree<br>○ Neutral<br>○ Somewhat disagree<br>○ Disagree<br>○ Strongly disagree |
| SUSmbv | I am worried about getting sick from a mosquito-borne virus in my country of residence. | ○ Strongly agree<br>○ Agree<br>○ Somewhat agree<br>○ Neutral<br>○ Somewhat disagree<br>○ Disagree<br>○ Strongly disagree |
| SUSres | I am at risk of getting infected with a mosquito-borne virus in my country of residence. | ○ Strongly agree<br>○ Agree<br>○ Somewhat agree<br>○ Neutral<br>○ Somewhat disagree<br>○ Disagree |

| | | |
|---|---|---|
| | | ○ Strongly disagree |
| SEVprobs | Getting sick with a mosquito-borne virus may result in hospitalisation. | ○ Strongly agree<br>○ Agree<br>○ Somewhat agree<br>○ Neutral<br>○ Somewhat disagree<br>○ Disagree<br>○ Strongly disagree |
| SEVdeadly | People can die from a mosquito-borne virus infection. | ○ Strongly agree<br>○ Agree<br>○ Somewhat agree<br>○ Neutral<br>○ Somewhat disagree<br>○ Disagree<br>○ Strongly disagree |
| SEVqual | Getting sick with a mosquito-borne virus can reduce your ability to do daily tasks. | ○ Strongly agree<br>○ Agree<br>○ Somewhat agree<br>○ Neutral<br>○ Somewhat disagree<br>○ Disagree<br>○ Strongly disagree |
| ControlA | Please select "Somewhat agree" as your answer Choice. | ○ Strongly agree<br>○ Agree<br>○ Somewhat agree<br>○ Neutral<br>○ Somewhat disagree<br>○ Disagree<br>○ Strongly disagree |

**Section 6. Awareness about Prevention Measures**

**This section aims to understand your confidence in preventing yourself from being bitten by a mosquito and how you would like to be reminded about using prevention measures. These questions are about your country of residence.**

<u>Questions within this section are randomized.</u>

| **Question code** | **Question** | **Answer Choices** |
|---|---|---|
| SEbest | I know which prevention measures are best to use against mosquito bites. | ○ Strongly agree<br>○ Agree<br>○ Somewhat agree<br>○ Neutral<br>○ Somewhat disagree<br>○ Disagree<br>○ Strongly disagree |
| SEinfo | I know where to find information about prevention measures against mosquito bites. | ○ Strongly agree<br>○ Agree<br>○ Somewhat agree<br>○ Neutral<br>○ Somewhat disagree |

| | | |
|---|---|---|
| | | ○ Disagree<br>○ Strongly disagree |
| SEbreedid | I am confident I can identify mosquito breeding sites. | ○ Strongly agree<br>○ Agree<br>○ Somewhat agree<br>○ Neutral<br>○ Somewhat disagree<br>○ Disagree<br>○ Strongly disagree |
| SEbreedrem | I am confident I can remove mosquito breeding sites in and around my house during mosquito season (March to September). | ○ Strongly agree<br>○ Agree<br>○ Somewhat agree<br>○ Neutral<br>○ Somewhat disagree<br>○ Disagree<br>○ Strongly disagree |
| CUEout | During the summer, going outside (hiking in nature, camping, picnics, gardening) reminds me to use prevention measures against mosquitoes. | ○ Strongly agree<br>○ Agree<br>○ Somewhat agree<br>○ Neutral<br>○ Somewhat disagree<br>○ Disagree<br>○ Strongly disagree |
| CUEmosq | Mosquitoes in and around my house at night remind me to use prevention measures against mosquitoes. | ○ Strongly agree<br>○ Agree<br>○ Somewhat agree<br>○ Neutral<br>○ Somewhat disagree<br>○ Disagree<br>○ Strongly disagree |
| CUEnotif | Getting news alerts about mosquito-borne virus cases in my area would remind me to use prevention measures. | ○ Strongly agree<br>○ Agree<br>○ Somewhat agree<br>○ Neutral<br>○ Somewhat disagree<br>○ Disagree<br>○ Strongly disagree |
| ControlB | Please select "Disagree" as your answer Choice. | ○ Strongly agree<br>○ Agree<br>○ Somewhat agree<br>○ Neutral<br>○ Somewhat disagree<br>○ Disagree<br>○ Strongly disagree |
| **Section 7. Background Information**<br><br>**In this section of the survey, we ask some general questions about what could influence your thoughts on mosquitoes and mosquito-borne viruses.** | | |
| **Question code** | **Question** | **Answer Choices** |

| | | |
|---|---|---|
| DEMmbvinf | I know someone who has gotten sick from a mosquito-borne virus. | ○ Yes<br>○ No<br>○ I do not know. |
| DEMgp | If I have flu-like symptoms, I contact my general practitioner. *<br><br>**(We would like to know when you would contact your general practitioner. This question is not specifically about mosquito-borne viruses but flu-like symptoms you might experience in general, not related to COVID-19.)* | ○ Strongly agree<br>○ Agree<br>○ Somewhat agree<br>○ Neutral<br>○ Somewhat disagree<br>○ Disagree<br>○ Strongly disagree |
| DEMinfo | Have you read or heard any information about mosquito-borne viruses from any of the following sources recently?<br><br>(Select all that apply) | ❑ Health professionals<br>❑ Government website<br>❑ Social media (e.g. Instagram, Twitter, Facebook, YouTube, online news articles)<br>❑ Family and Friends<br>❑ Educational institutes (e.g. school and universities)<br>❑ Institutional websites (e.g. WHO, ECDC, WOAH)<br>❑ Television and News Channels<br>❑ Print Newspaper<br>❑ Radio<br>❑ Communication campaign<br>❑ None of the above |
| DEMsearch | Where would you like to find or receive information about mosquitoes and mosquito-borne viruses? | ❑ Health professionals<br>❑ Government website<br>❑ Social media (e.g. Instagram, Twitter, Facebook, YouTube, online news articles)<br>❑ Family and Friends<br>❑ Educational institutes (e.g. school and universities)<br>❑ Institutional websites (e.g. WHO, ECDC, WOAH)<br>❑ Television and News Channels |

| | | |
|---|---|---|
| | | ❑ Print Newspaper<br>❑ Radio<br>❑ Communication campaign<br>❑ I am not interested in receiving information<br>❑ Other [ open answer ] |
| **Section 8. Travel History**<br><br>**This section is about your travel history.** | | |
| **Question code** | **Question** | **Answer Choices** |
| DEMtravel | Where have you been on holiday in the past two years (2020-2022)? | ❑ I have not been on holiday outside my country of residence. > Conditional. If selected, go to DEMadvice<br>❑ I have been on holiday within Europe.> Conditional. If selected, go to DEMadvice<br>❑ I have been on holiday outside of Europe. > Conditional. If selected, go to DEMadvice |
| DEMeu | (Conditional)<br><br>Please select which regions and countries within Europe you have been on holiday to in the past 2 years (2020-2022). | ❑ Belgium<br>❑ Canary islands, Azores, Madeira<br>❑ Denmark<br>❑ Germany<br>❑ Finland<br>❑ France (including Corsica)<br>❑ Greece and/or Greek islands<br>❑ Italy (including Sicily, Sardinia)<br>❑ Ireland<br>❑ Iceland<br>❑ Luxembourg<br>❑ Norway<br>❑ Eastern Europe (includes Hungary, Czech Republic, Slovenia, Poland, Croatia)<br>❑ Austria<br>❑ Portugal<br>❑ Spain (including Menorca, Mallorca, Ibiza)<br>❑ Turkey<br>❑ Sweden<br>❑ Switzerland<br>❑ Other: [open answer] |
| DEMnoneu | (Conditional) | ❑ Northern Africa<br>❑ Sub-Saharan Africa (includes Western, |

| | | |
|---|---|---|
| | To which regions outside of Europe, have you been on holiday in the past 2 years (2020-2022)? *See below for a map of the regions.<br>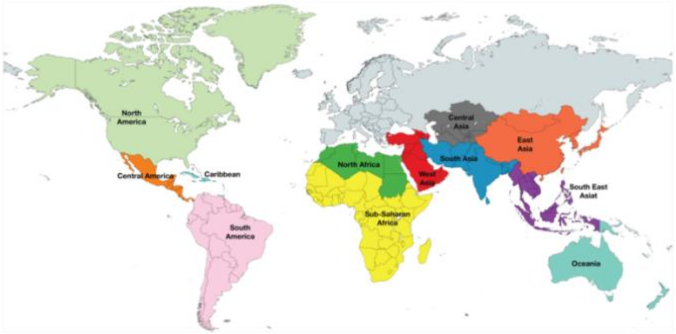<br> | Middle, Eastern and Southern Africa)<br>❑ Northern America<br>❑ Caribbean<br>❑ Central America<br>❑ South America<br>❑ Central Asia<br>❑ Eastern Asia<br>❑ South Eastern Asia<br>❑ Southern Asia<br>❑ Western Asia<br>❑ Oceania |
| DEMadvice | Have you ever received travel-related advice about any mosquito-borne viruses?<br><br>* Malaria is a parasitic disease transmitted by mosquitoes. | ❑ Yes<br>❑ No<br>❑ I am not sure if I have ever received travel-related health advice about any mosquito-borne viruses.<br>❑ No, but I have received travel-related health advice about malaria* |
| **Section 9. Demographics**<br><br>**In this final section of the survey, we ask some general questions about your demographic characteristics.** | | |
| **Question code** | **Question** | **Answer Choices** |
| DEMage | How old are you? | Numeric open answer [18 to 100] |
| DEMgender | What is your gender? | ○ Man<br>○ Woman<br>○ Other [open answer]<br>○ I prefer not to disclose. |
| DEMeduc | What is the highest degree or level of education you have completed? | ○ Primary School<br>○ Secondary School<br>○ Post-Secondary (includes technical/community college and university)<br>○ None of the above |

| | | |
|---|---|---|
| DEMoccustatus | What is your current employment status? | ○ Employed > Conditional. If selected, go to DEMindustry<br>○ Student > Conditional. If selected, go to DEMindustry<br>○ Homemaker<br>○ (Currently) Unemployed<br>○ Retired |
| DEMindustry | (Conditional)<br><br>Which of the following categories best describes your industry you primarily work in or study (regardless of your actual position)? | ❑ Architecture, Engineering, Computer and Mathematical<br>❑ Armed Forces and Military<br>❑ Arts, Design, Entertainment, Sports and Media<br>❑ Construction and Extraction, Building and Grounds Cleaning and Maintenance<br>❑ Education, Training, Library, Community and Social Service<br>❑ Farming, Fishing and Forestry<br>❑ Healthcare Practitioners, Technical support and Healthcare Support<br>❑ Installation, Maintenance, Repairs, Crafts and Related Trades<br>❑ Legal Services, Business and Financial Operations<br>❑ Management, Office and Administrative Support<br>❑ Scientific Technicians and Professionals<br>❑ Service and Sales, Hospitality and Related (e.g. Food Preparation, Personal Care, Real Estate)<br>❑ Transportation and Materials Moving |
| DEMres | What is your country of residence? | ○ England<br>○ Wales |

| | | |
|---|---|---|
| | | ○ Scotland<br>○ Northern Ireland<br>○ Other [open answer] |
| DEMarea | Do you live in an urban or rural area? | ○ Urban<br>○ Rural<br>○ I do not know. |
| DEMprovince | In which province do you live? | ○ Dropdown list |
| DEMcs | Have you ever used any of the following (citizen science) websites or mobile applications? | ❑ Mosquito Alert<br>❑ Muggenradar<br>❑ Mückenatlas<br>❑ ZanzaMapp<br>❑ I have not used any of these citizen science websites or applications. |
| End of the survey | | |

## Final Survey Version - Dutch

<table>
<tr><td colspan="3">Eerste pagina vragenlijst<br><br>Achtergrondinformatie over het doel van de vragenlijst<br><br>Het doel van deze studie is om te begrijpen wat mensen weten en wat hun percepties zijn voor muggen en door muggen overdraagbare virussen in het land waar je woont. Ook willen we graag weten wat voor voorzorgsmaatregelen mensen nemen om muggenbeten te voorkomen.<br><br>Deze vragenlijst is bedoeld voor mensen van 18 jaar en ouder die in Nederland of Spanje wonen. Het invullen van de vragenlijst zou niet langer dan 15 minuten moeten duren. Deelname is vrijwillig en alle antwoorden blijven anoniem. Door op volgende te klikken ga je akkoord met deelname aan dit onderzoek en geef je ons toestemming om jouw anonieme antwoorden op te slaan voor analyse.<br><br>Je deelname helpt ons enorm in ons onderzoek. Alvast heel erg bedankt voor je tijd!</td></tr>
<tr><td colspan="3">Onderdeel 1. Jouw woonomgeving en dagelijks leven, ervaringen met muggen<br><br>-Omschrijving onderdeel -<br><br>Er zijn heel veel verschillende soorten muggen. Sommige muggensoorten bijten mensen en worden daarom ook wel steekmuggen genoemd. In deze vragenlijst hebben we het over steekmuggen, maar in de rest van de vragenlijst noemen we ze kortweg muggen. Deze vragen gaan over jouw ervaringen in- en rondom je huis.</td></tr>
<tr><td>Vraag Code</td><td>Vraag</td><td>Antwoord optie(s)</td></tr>
<tr><td>EXage</td><td>Ben je 18 jaar of ouder?</td><td>○ Ja> conditioneel, einde vragenlijst vanwege exclusiecriteria</td></tr>
</table>

| | | |
|---|---|---|
| | | ○ Nee |
| DEMout | Heeft je woning een buitenruimte die je gebruikt (zoals een tuin, balkon of dakterras)? | ○ Ja<br>○ Nee |
| DEMactive | Ik ervaar last van muggen tijdens het doen van de volgende activiteiten* tijdens het muggenseizoen (maart tot en met september):<br><br>Selecteer alle antwoorden die van toepassing zijn<br><br>* Deze vraag gaat over het doen van deze activiteiten in het land waar je woont. | ❑ Tijd doorbrengen in- en rondom mijn huis (zoals in de tuin, op het balkon) > conditioneel, als geselecteerd DEMactive1<br>❑ Buiten sporten/ bewegen (zoals, voetbal of hardlopen) > conditioneel, als geselecteerd DEMactive2<br>❑ Buiten zijn voor vrijetijdsbesteding (picknicken, kamperen, wandelen) > conditioneel, als geselecteerd DEMactive3<br>❑ Werken > conditioneel, als geselecteerd DEMactive4<br>❑ Slapen > conditioneel, als geselecteerd DEMactive5<br>❑ Reizen van- en naar mijn werk of studie > conditioneel, als geselecteerd DEMactive6<br>❑ Anders<br>❑ Ik ervaar geen last van steekmuggen tijdens de bovenstaande activiteiten |
| DEMactive1 | (Conditioneel)<br><br>Hoe vaak irriteren muggen je wanneer je tijd doorbrengt in- en rondom je huis (zoals in de tuin, op het balkon) tijdens het muggenseizoen (maart tot en met september)? | ○ Heel vaak<br>○ Vaak<br>○ Af en toe<br>○ Bijna nooit |
| DEMactive2 | (Conditioneel)<br><br>Hoe vaak irriteren muggen je wanneer je buiten sport of beweegt (zoals voetbal of hardlopen) tijdens het muggenseizoen (maart tot en met september)? | ○ Heel vaak<br>○ Vaak<br>○ Af en toe<br>○ Bijna nooit |
| DEMactive3 | (Conditioneel)<br><br>Hoe vaak irriteren muggen je wanneer je buiten bent voor vrijetijdsbesteding (zoals picknicken, kamperen, wandelen) tijdens het muggenseizoen (maart tot en met september)? | ○ Heel vaak<br>○ Vaak<br>○ Af en toe<br>○ Bijna nooit |
| DEMactive4 | (Conditioneel)<br><br>Hoe vaak irriteren muggen je wanneer je aan het werk bent tijdens het muggenseizoen (maart tot en met september)? | ○ Heel vaak<br>○ Vaak<br>○ Af en toe<br>○ Bijna nooit |

| | | |
|---|---|---|
| DEMactive5 | (Conditioneel)<br><br>Hoe vaak irriteren muggen je wanneer je slaapt tijdens het muggenseizoen (maart tot en met september)? | ○ Heel vaak<br>○ Vaak<br>○ Af en toe<br>○ Bijna nooit |
| DEMactive6 | (Conditioneel)<br><br>Hoe vaak irriteren muggen je wanneer je reist van- en naar je werk of studie (woon-werkverkeer) tijdens het muggenseizoen (maart tot en met september)? | ○ Heel vaak<br>○ Vaak<br>○ Af en toe<br>○ Bijna nooit |
| **Onderdeel 2. Stellingen over muggen en door muggen overdraagbare virussen**<br><br>**Dit deel van de vragenlijst bevat stellingen over muggen, muggen broedplaatsen en door muggen overdraagbare virussen.**<br><br>De vragen in dit onderdeel zijn gerandomiseerd. | | |
| **Vraag code** | **Vraag** | **Antwoord optie(s)** |
| KNbite | Muggen bijten mensen alleen overdag | ○ Ja<br>○ Nee<br>○ Ik weet het niet |
| KNbreed | In tuinen kunnen muggen eitjes leggen in:<br><br>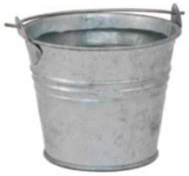 A. Stilstaand water in een emmer<br>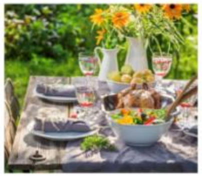 B. Etensresten op de tuintafel<br>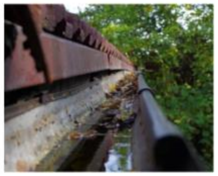 C. Stilstaand water in dakgoten<br><br>(Selecteer alle antwoorden die van toepassing zijn) | ❑ A. Stilstaand water in een emmer<br>❑ B. Etensresten op de tuintafel<br>❑ C. Stilstaand water in dakgoten<br>❑ Geen van de bovenstaande antwoorden<br>❑ Ik weet het niet |
| KNvirus | De volgende virussen worden (vooral) verspreid door muggen:<br><br>(Selecteer alle antwoorden die van toepassing zijn) | ❑ West Nijl virus<br>❑ Chikungunya virus<br>❑ Zika virus<br>❑ Influenza (griep)<br>❑ Dengue Virus<br>❑ Humaan Immunodeficiëntievirus (HIV)<br>❑ Mazelen virus<br>❑ Geen van de bovenstaande antwoorden<br>❑ Ik weet het niet |
| KNroute | Een persoon zou een door muggen overdraagbaar virus kunnen krijgen als die persoon: | ❑ Gebeten wordt door een besmette mug<br>❑ Een besmet persoon aanraakt<br>❑ Een besmet dier |

| | | |
|---|---|---|
| | (Selecteer alle antwoorden die van toepassing zijn) | ❑ Besmet raakt via de lucht<br>❑ Seksueel contact heeft met een besmet persoon<br>❑ Geen van de bovenstaande antwoorden<br>❑ Ik weet het niet. |

**Onderdeel 3. Voorzorgsmaatregelen tegen muggen**

**Dit deel van de vragenlijst gaat over voorzorgsmaatregelen tegen muggen. Het gaat om jouw ervaringen in- en rondom je huis.**

**In het vorige deel hebben we wat vragen gesteld over broedplaatsen. We willen je graag wat meer informatie geven over wat muggenbroedplaatsen zijn. Muggen kunnen eitjes leggen in stilstaand water, bijvoorbeeld in emmers of gieters in je tuin, water in verstopte dakgoten of in een regenton die niet is afgedekt.**

**Muggeneitjes groeien uit tot volwassen muggen in ongeveer 14 dagen en hebben daarvoor water nodig. Als je stilstaand water dus elke week weggiet kan je voorkomen dat de muggeneitjes uitgroeien tot volwassen muggen. Dit kan het aantal muggen rondom je huis verminderen.**

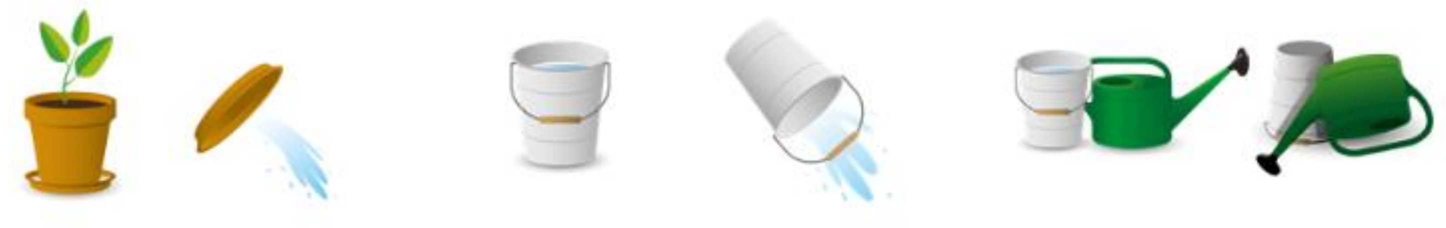

**bron NVWA**

| Vraag code | Vraag | Antwoord optie(s) |
|---|---|---|
| PMuse | Selecteer alle voorzorgsmaatregelen die je neemt tegen muggen:<br><br>(Selecteer alle antwoorden die van toepassing zijn) | ❑ Het dragen van lange mouwen en een lange broek > Conditioneel. Als geselecteerd ga naar PMreasonY<br>❑ Insectenspray gebruiken op mijn lichaam (zoals DEET) > Conditioneel. Als geselecteerd ga naar PMreasonY<br>❑ Een elektrische (vliegen)mepper gebruiken > Conditioneel. Als geselecteerd ga naar PMreasonY<br>❑ Een elektrische ventilator of airconditioning gebruiken > Conditioneel. Als geselecteerd ga naar PMreasonY<br>❑ Een anti-muggen(geur) stekker gebruiken > Conditioneel. Als geselecteerd ga naar PMreasonY |

| | | |
|---|---|---|
| | | ❑ Horren plaatsen voor deuren en/of ramen > Conditioneel. Als geselecteerd ga naar PMreasonY<br>❑ Slapen met slaapkamerraam(en) gesloten > Conditioneel. Als geselecteerd ga naar PMreasonY<br>❑ Slapen onder een klamboe (net tegen muggen) > Conditioneel. Als geselecteerd ga naar PMreasonY<br>❑ Natuurlijke methoden gebruiken: knoflook, kruiden en planten > Conditioneel. Als geselecteerd ga naar PMreasonY<br>❑ Verwijderen van stilstaand water in- en rondom mijn huis > Conditioneel. Als geselecteerd ga naar PMreasonY<br>❑ Anders > conditioneel. Als geselecteerd ga naar PPreasonN<br>❑ Geen |
| PMreasonN | (Conditioneel) Om welke reden(en) neem je geen voorzorgsmaatregelen tegen muggen?<br><br>(Selecteer alle antwoorden die van toepassing zijn) | ❑ Er zijn geen muggen waar ik woon<br>❑ Voorzorgsmaatregelen zijn vervelend<br>❑ Voorzorgsmaatregelen kosten (te) veel tijd<br>❑ Muggen bijten mij niet<br>❑ Voorzorgsmaatregelen zijn te duur<br>❑ Anders |
| PMreasonY | (Conditioneel) Om welke reden(en) neem je deze voorzorgsmaatregelen tegen muggen?<br><br>(Selecteer alle antwoorden die van toepassing zijn) | ❑ Om minder gebeten te worden<br>❑ Om het aantal muggen in- en om mijn huis te verminderen<br>❑ Om de kans op het krijgen van een door muggen overdraagbaar virus te verminderen<br>❑ Anders |
| RESpp | Wie denk je dat er verantwoordelijk zou moeten zijn voor het informeren van het publiek over voorzorgsmaatregelen tegen muggen?<br><br>(Selecteer alle antwoorden die van toepassing zijn) | ❑ De overheid, inclusief ministerie van volksgezondheid<br>❑ Zorgmedewerkers (zoals dokters en verpleegkundigen)<br>❑ Burgers zelf |
| RESpbreed | Ik denk dat ik voornamelijk zelf verantwoordelijk ben voor het verwijderen van muggenbroedplaatsen in- en rondom mijn huis | ○ Helemaal eens<br>○ Eens<br>○ Enigszins eens<br>○ Neutraal |

| | | |
|---|---|---|
| | | o Enigszins oneens<br>o Oneens<br>o Helemaal oneens |
| RESgovbreed | Ik denk dat de overheid* voornamelijk verantwoordelijk is voor het verwijderen van muggenbroedplaatsen in mijn buurt.<br><br>(lokale gemeente, ministeries van volksgezondheid en hun gerelateerde instituten) | o Helemaal eens<br>o Eens<br>o Enigszins eens<br>o Neutraal<br>o Enigszins oneens<br>o Oneens<br>o Helemaal oneens |
| **Onderdeel 4. Jouw mening over voorzorgsmaatregelen**<br><br>**In dit deel van de vragenlijst stellen we vragen over wat jij denkt over het gebruiken van (bepaalde) voorzorgsmaatregelen tegen muggen(beten).**<br><br><u>De vragen in dit onderdeel zijn gerandomiseerd</u> | | |
| **Vraag code** | **Vraag** | **Antwoord optie(s)** |
| BENbites | Het gebruiken van insectenspray (zoals DEET) op de huid voorkomt muggenbeten.<br><br>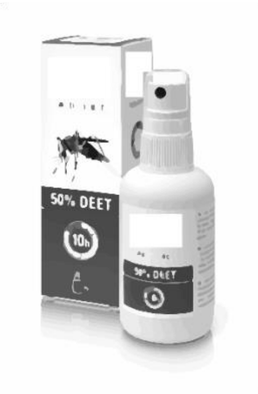 | o Helemaal eens<br>o Eens<br>o Enigszins eens<br>o Neutraal<br>o Enigszins oneens<br>o Oneens<br>o Helemaal oneens |
| BENsafe | Insectenspray voor op de huid (zoals DEET) is veilig om te gebruiken. | o Helemaal eens<br>o Eens<br>o Enigszins eens<br>o Neutraal<br>o Enigszins oneens<br>o Oneens<br>o Helemaal oneens |

| | | |
|---|---|---|
| | 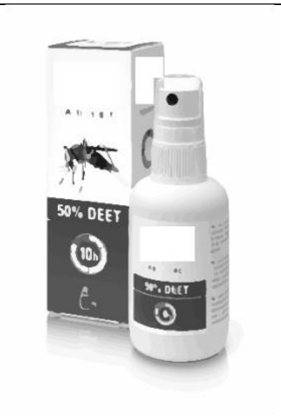 | |
| BENprev | Als ik voorzorgsmaatregelen neem voorkom ik dat ik door muggen wordt gebeten | ○ Helemaal eens<br>○ Eens<br>○ Enigszins eens<br>○ Neutraal<br>○ Enigszins oneens<br>○ Oneens<br>○ Helemaal oneens |
| BARannoy | Voorzorgsmaatregelen nemen tegen muggen is vervelender dan de muggen zelf. | ○ Helemaal eens<br>○ Eens<br>○ Enigszins eens<br>○ Neutraal<br>○ Enigszins oneens<br>○ Oneens<br>○ Helemaal oneens |
| BARtime | Voorzorgsmaatregelen nemen tegen muggen kost te veel tijd. | ○ Helemaal eens<br>○ Eens<br>○ Enigszins eens<br>○ Neutraal<br>○ Enigszins oneens<br>○ Oneens<br>○ Helemaal oneens |

**Onderdeel 5: Muggen en door muggen overdraagbare virussen**

**Dit deel van de vragenlijst gaat over jouw ervaringen in- en rondom je huis in het land waar je woont. We willen graag begrijpen wat jouw kijk is op muggen en door muggen overdraagbare virussen.**

<u>De vragen in dit onderdeel zijn gerandomiseerd.</u>

| Vraag code | Vraag | Antwoord optie |
|---|---|---|
| SUSmosq | Ik woon in een buurt waar veel muggen zijn. | ○ Helemaal eens<br>○ Eens<br>○ Enigszins eens<br>○ Neutraal<br>○ Enigszins oneens<br>○ Oneens<br>○ Helemaal oneens |
| SUSbite | De kans om in het land waar ik woon gebeten te worden door een mug in is groot. | ○ Helemaal eens<br>○ Eens<br>○ Enigszins eens<br>○ Neutraal<br>○ Enigszins oneens<br>○ Oneens<br>○ Helemaal oneens |
| SUSmbv | Ik maak me zorgen dat ik in het land waar ik woon ziek wordt van een door muggen overdraagbaar virus. | ○ Helemaal eens<br>○ Eens<br>○ Enigszins eens<br>○ Neutraal<br>○ Enigszins oneens<br>○ Oneens<br>○ Helemaal oneens |
| SUSres | Ik loop het risico besmet te raken met een door muggen overdraagbaar virus in het land waar ik woon. | ○ Helemaal eens<br>○ Eens<br>○ Enigszins eens<br>○ Neutraal<br>○ Enigszins oneens<br>○ Oneens<br>○ Helemaal oneens |
| SEVprobs | Ziek worden van een door muggen overdraagbaar virus kan een ziekenhuisopname als gevolg hebben. | ○ Helemaal eens<br>○ Eens<br>○ Enigszins eens<br>○ Neutraal<br>○ Enigszins oneens<br>○ Oneens<br>○ Helemaal oneens |
| SEVdeadly | Mensen kunnen overlijden aan een besmetting met een door muggen overdraagbaar virus. | ○ Helemaal eens<br>○ Eens<br>○ Enigszins eens |

| | | |
|---|---|---|
| | | ○ Neutraal<br>○ Enigszins oneens<br>○ Oneens<br>○ Helemaal oneens |
| SEVqual | Ziek worden van een door muggen overdraagbaar virus kan je vermogen om dagelijkse taken uit te voeren verminderen. | ○ Helemaal eens<br>○ Eens<br>○ Enigszins eens<br>○ Neutraal<br>○ Enigszins oneens<br>○ Oneens<br>○ Helemaal oneens |
| ControlA | Selecteer alsjeblieft “enigszins eens” als je antwoordoptie | ○ Helemaal eens<br>○ Eens<br>○ Enigszins eens<br>○ Neutraal<br>○ Enigszins oneens<br>○ Oneens<br>○ Helemaal oneens |
| **Onderdeel 6. Bewustzijn van voorzorgsmaatregelen**<br><br>**Met deze vragen willen we begrijpen hoe zeker je bent dat je kan voorkomen dat je gebeten wordt door muggen, en hoe je eventueel herinnerd zou kunnen worden aan het nemen van voorzorgsmaatregelen. De vragen in dit onderdeel gaan over het land waar je woont.**<br><br><u>De vragen in dit onderdeel zijn gerandomiseerd.</u> | | |
| **Vraag code** | **Vraag** | **Antwoord optie(s)** |
| SEbest | Ik weet welke voorzorgsmaatregelen ik het beste kan nemen tegen muggenbeten. | ○ Helemaal eens<br>○ Eens<br>○ Enigszins eens<br>○ Neutraal<br>○ Enigszins oneens<br>○ Oneens<br>○ Helemaal oneens |
| SEinfo | Ik weet waar ik informatie kan vinden over voorzorgsmaatregelen tegen muggenbeten. | ○ Helemaal eens<br>○ Eens<br>○ Enigszins eens<br>○ Neutraal<br>○ Enigszins oneens<br>○ Oneens<br>○ Helemaal oneens |

| | | |
|---|---|---|
| SEbreedid | Ik ben er zeker van dat ik muggenbroedplaatsen kan identificeren. | ○ Helemaal eens<br>○ Eens<br>○ Enigszins eens<br>○ Neutraal<br>○ Enigszins oneens<br>○ Oneens<br>○ Helemaal oneens |
| SEbreedrem | Ik ben er zeker van dat ik muggen broedplaatsen in- en om mijn huis kan verwijderen tijdens het muggenseizoen (maart tot en met september). | ○ Helemaal eens<br>○ Eens<br>○ Enigszins eens<br>○ Neutraal<br>○ Enigszins oneens<br>○ Oneens<br>○ Helemaal oneens |
| CUEout | Naar buiten gaan in de zomer (zoals wandelingen in de natuur, kamperen, picknicks, tuinieren) herinnert mij eraan om voorzorgsmaatregelen te nemen tegen muggen. | ○ Helemaal eens<br>○ Eens<br>○ Enigszins eens<br>○ Neutraal<br>○ Enigszins oneens<br>○ Oneens<br>○ Helemaal oneens |
| CUEmosq | Muggen in- en om mijn huis ‘s nachts herinneren me eraan om voorzorgsmaatregelen te nemen tegen muggen. | ○ Helemaal eens<br>○ Eens<br>○ Enigszins eens<br>○ Neutraal<br>○ Enigszins oneens<br>○ Oneens<br>○ Helemaal oneens |
| CUEnotif | Als ik nieuwsberichten ontvang over ziektegevallen door een door muggen overdraagbaar virus in mijn regio, zou ik eraan herinnerd worden om voorzorgsmaatregelen te nemen. | ○ Helemaal eens<br>○ Eens<br>○ Enigszins eens<br>○ Neutraal<br>○ Enigszins oneens<br>○ Oneens<br>○ Helemaal oneens |
| ControlB | Selecteer alsjeblieft “oneens” als je antwoordoptie | ○ Helemaal eens<br>○ Eens<br>○ Enigszins eens<br>○ Neutraal |

| | | |
|---|---|---|
| | | ○ Enigszins oneens<br>○ Oneens<br>○ Helemaal oneens |
| **Onderdeel 7: Achtergrondinformatie**<br><br>**In dit deel van de vragenlijst stellen we wat algemene vragen over dingen die jouw mening over muggen en door muggen overdraagbare virussen kunnen beïnvloeden.** | | |
| **Vraag code** | **Vraag** | **Antwoord optie(s)** |
| DEMmbvinf | Ik ken iemand die ziek is geworden van een door muggen overdraagbaar virus. | ○ Ja<br>○ Nee<br>○ Ik weet het niet |
| DEMgp | Als ik griepachtige klachten heb, neem ik contact op met mijn huisarts<br><br>*(We willen graag weten wanneer je contact zou opnemen met je huisarts. Deze vraag gaat niet specifiek over* door steekmuggen overdraagbare virussen*, maar over griepklachten die je in het algemeen kan ervaren, niet gerelateerd aan COVID-19)* | ○ Helemaal eens<br>○ Eens<br>○ Enigszins eens<br>○ Neutraal<br>○ Enigszins oneens<br>○ Oneens<br>○ Helemaal oneens |
| DEMinfo | Heb je kort geleden nog informatie gelezen of gehoord over muggen of door muggen overdraagbare virussen via één of meerdere van deze bronnen?<br><br>(Selecteer alle antwoorden die van toepassing zijn) | ❑ Zorgmedewerkers<br>❑ Overheidswebsites<br>❑ Sociale media (b.v. Instagram, Twitter, Facebook, YouTube, online nieuws artikelen)<br>❑ Familie en vrienden<br>❑ Onderwijsinstellingen (bijvoorbeeld school of universiteiten)<br>❑ Institutionele websites (b.v. WHO, ECDC, WOAH)<br>❑ Televisie- en nieuwskanalen<br>❑ Papieren krant<br>❑ Radio<br>❑ Communicatiecampagne<br>❑ Geen van bovenstaande bronnen |
| DEMsearch | Waar zou je graag informatie willen vinden of ontvangen over muggen of door muggen overdraagbare virussen?<br><br>(Selecteer alle antwoorden die van toepassing zijn) | ❑ Zorgmedewerkers<br>❑ Overheidswebsites<br>❑ Sociale media (b.v. Instagram, Twitter, Facebook, YouTube, online nieuws artikelen)<br>❑ Familie en vrienden |

| | | |
|---|---|---|
| | | ❑ Onderwijsinstellingen (bijvoorbeeld school of universiteiten)<br>❑ Institutionele websites (b.v. WHO, ECDC, WOAH)<br>❑ Televisie- en nieuwskanalen<br>❑ Papieren krant<br>❑ Radio<br>❑ Communicatiecampagne<br>❑ Ik wil geen informatie ontvangen<br>❑ Anders [ open antwoord ] |
| **Onderdeel 8. Reizen**<br><br>**Deze vragen gaan over reizen en vakanties.** | | |
| **Vraag code** | **Vraag** | **Antwoord optie(s)** |
| DEMtravel | Waar ben je de afgelopen twee jaar (2020-2022) op vakantie geweest?<br><br>(Selecteer alle antwoorden die van toepassing zijn) | ❑ Ik ben niet buiten het land waar ik woon op vakantie geweest > *conditioneel, als geselecteerd ga naar DEMadvice*<br>❑ Ik ben binnen Europa op vakantie geweest> *conditioneel, , als geselecteerd ga naar DEMeu*<br>❑ Ik ben buiten Europa op vakantie geweest *> conditioneel, , als geselecteerd ga naar DEMnoneu* |
| DEMeu | (Conditioneel) Selecteer alsjeblieft in welke regio's en/of landen binnen Europa, je de afgelopen twee jaar (2020-2022) op vakantie bent geweest?<br><br>(Selecteer alle antwoorden die van toepassing zijn) | ❑ België<br>❑ Canarische eilanden, Azoren en/of Madeira<br>❑ Denemarken<br>❑ Duitsland<br>❑ Finland<br>❑ Frankrijk (inclusief Corsica)<br>❑ Griekenland en/of de Griekse eilanden<br>❑ Italië (inclusief Sicilië en Sardinië)<br>❑ Ierland<br>❑ IJsland<br>❑ Luxemburg<br>❑ Noorwegen<br>❑ Oost-Europa (Hongarije, Tsjechië, Slovenië, Polen, Kroatië)<br>❑ Oostenrijk<br>❑ Portugal |

| | | |
|---|---|---|
| | | ❑ Spanje (inclusief Menorca, Mallorca en Ibiza)<br>❑ Turkije<br>❑ Verenigd Koninkrijk (Engeland, Schotland, Wales, Noord-Ierland)<br>❑ Zweden<br>❑ Zwitserland<br>❑ Andere [ open antwoord ] |
| DEMnoneu | (Conditioneel) Selecteer alsjeblieft in welke regio's en/of landen buiten Europa, je de afgelopen twee jaar (2020-2022) op vakantie bent geweest?<br><br>(Selecteer alle antwoorden die van toepassing zijn)<br><br>*Op de kaart hieronder staan de verschillende regio's aangegeven<br><br>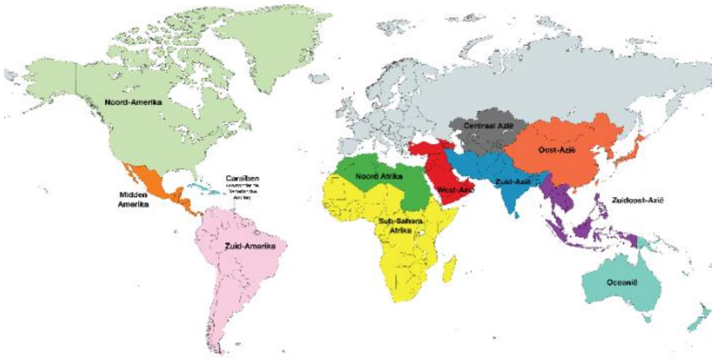 | ❑ Noord Afrika<br>❑ Sub-Sahara Afrika (waaronder West-, Midden-, Oost- en Zuidelijk Afrika)<br>❑ Noord-Amerika<br>❑ Caraïben (waaronder de Nederlandse Antillen)<br>❑ Midden-Amerika<br>❑ Zuid-Amerika<br>❑ Centraal Azië<br>❑ Oost-Azië<br>❑ Zuidoost-Azië<br>❑ Zuid-Azië<br>❑ West-Azië<br>❑ Oceanië |
| DEMadvice | Heb je ooit reis gerelateerd gezondheidsadvies gekregen over door muggen overdraagbare virussen?<br><br>(*malaria is ziekte veroorzaakt door een door muggen overdraagbare parasiet) | ○ Ja<br>○ Nee<br>○ Ik weet niet zeker of ik ooit reis gerelateerd gezondheidsadvies heb gekregen over door steekmuggen overdraagbare virussen.<br>○ Nee, maar ik heb wel reis gerelateerd gezondheidsadvies gekregen over malaria. * |
| **Onderdeel 9. Demografische kenmerken** | | |

**In dit laatste deel van de vragenlijst vragen we wat algemene gegevens.**

| **Q code** | **Vraag** | **Antwoord optie** |
|---|---|---|
| DEMage | Hoe oud ben je? | Open, Numeriek [18 tot 100] |
| DEMgender | Wat is je gender? | ○ Man<br>○ Vrouw<br>○ Anders [open antwoord]<br>○ Vertel ik liever niet |
| DEMeduc | Wat is je hoogst afgeronde opleiding? | ○ Basisschool<br>○ Middelbare school<br>○ Hoger onderwijs (MBO; HBO; Universiteit en hoger)<br>○ Geen van de bovenstaande |
| DEMoccustatus | Wat is je huidige werksituatie? | ○ Werkende > conditioneel, als geselecteerd ga naar (DEMindustry)<br>○ Student > conditioneel, als geselecteerd ga naar (DEMindustry)<br>○ Huisvrouw/ -man<br>○ (Momenteel) werkloos<br>○ Gepensioneerd |
| DEMindustry | (Conditioneel) Welke van de volgende categorieën omschrijft de sector waarin je voornamelijk werkt of studeert het beste? (Onafhankelijk van je functie)? | ○ Architectuur, werkbouwtuigkunde, bouwkunde, ICT en wiskunde<br>○ Defensie (strijdkrachten en leger)<br>○ Kunst, design, entertainment, sport en media<br>○ Bouw en (grondstof)winning, reiniging en onderhoud van gebouwen en terreinen<br>○ Onderwijs, training, bibliotheek, sociaal-en maatschappelijke werk/ diensterverlening<br>○ Landbouw, visserij en bosbouw<br>○ Zorgverleners, technische ondersteuning en zorgondersteuning<br>○ Installatie, onderhoud, reparaties, ambachten en aanverwante beroepen<br>○ Juridische dienstverlening, zakelijke en financiële dienstverlening<br>○ Management, Office- en Administratieve Ondersteuning<br>○ Wetenschappelijke technici en professionals |

| | | |
|---|---|---|
| | | ○ Service en Verkoop, Horeca en gerelateerde beroepen (bijv. Voedselbereiding, Persoonlijke Verzorging, Vastgoed)<br>○ Transport en logistiek |
| DEMres | In welk land woon je? | ○ Nederland (inclusief de Nederlandse Antillen)<br>○ Andere [ open antwoord ] |
| DEMarea | Woon je in een stedelijk of landelijk gebied? | ○ Stedelijk<br>○ Landelijk<br>○ Weet ik niet |
| DEMprovince | In welke provincie woon je? | ○ Drenthe<br>○ Flevoland<br>○ Friesland<br>○ Gelderland<br>○ Groningen<br>○ Limburg<br>○ Noord-Brabant<br>○ Noord-Holland<br>○ Overijssel<br>○ Utrecht<br>○ Zeeland<br>○ Zuid-Holland<br>○ Nederlandse Antillen |
| DEMcs | Heb je ooit een van de volgende 'citizen science" mobiele applicaties (apps) of websites gebruikt? | ❑ Mosquito Alert<br>❑ Muggenradar<br>❑ Mückenatlas<br>❑ ZanzaMapp<br>❑ Ik heb geen van deze 'citizen science" mobiele applicaties(apps) of websites gebruikt. |
| Einde vragenlijst | | |

## Final Survey Version - Spanish

| |
|---|
| **Primera página de la encuesta** |

**Título de la encuesta**

**Información de antecedentes sobre el propósito de la investigación y el uso de las respuestas de la encuesta.**

***El propósito de esta encuesta es comprender mejor tus conocimientos y percepciones sobre los mosquitos y los virus transmitidos por mosquitos en tu país de residencia. El objetivo de esta encuesta también es comprender qué medidas preventivas actuales utilizas para evitar las picaduras de mosquitos y evitar enfermarse con los virus transmitidos por mosquitos. Esta encuesta no debe tomar más de 15 minutos.***

***Tu participación es completamente voluntaria y todas las respuestas permanecerán anónimas. Tus respuestas nos ayudarán mucho. ¡Muchísimas gracias por tu tiempo!***

**Sección 1. Tu Entorno y Experiencias con los Mosquitos**

Esta sección trata sobre tus experiencias con los mosquitos dentro y alrededor de tu hogar.

| **Código de pregunta** | **Pregunta** | **Opción de respuesta** |
|---|---|---|
| EXage | ¿Tienes 18 años o más? | ○ Sí > condicional, la encuesta finaliza debido a criterios de exclusión.<br>○ No |
| DEMout | ¿Tiene tu casa espacio exterior que uses (como un jardín, balcón o terraza)? | ○ Sí<br>○ No |
| DEMactive | Experimento molestias por mosquitos durante la temporada de mosquitos (de marzo a septiembre) durante las siguientes actividades | ❑ Pasando tiempo dentro y alrededor de mi casa de mi casa (en el jardín, en el balcón) > condicional. si se selecciona ir a DEMactive1<br>❑ Haciendo deporte o ejercicio en exteriores (por ejemplo, jugando el fútbol o corriendo) > condicional. si se selecciona ir a DEMactive2<br>❑ Estando afuera para actividades de ocio (p. ej., picnics, campamento, caminatas) > condicional. si se selecciona ir a DEMactive3<br>❑ Trabajando > condicional. si se selecciona ir a DEMactive4<br>❑ Durmiendo > condicional. si se selecciona ir a DEMactive5<br>❑ Viajando con fines educativos o laborales (trayectos hacía el trabajo) > condicional. si se selecciona ir a DEMactive6<br>❑ Otras<br>❑ No experimento molestias por mosquitos durante las actividades mencionadas anteriormente. |

| | | |
|---|---|---|
| DEMactive1 | (Condicional) ¿Con qué frecuencia te molestan los mosquitos cuando pasas tiempo dentro y alrededor de tu casa (jardín, balcón)? | ○ Muy frecuentemente<br>○ Frecuentemente<br>○ Ocasionalmente<br>○ Raramente |
| DEMactive2 | (Condicional) ¿Con qué frecuencia te molestan los mosquitos cuando haces deporte o ejercicio al aire libre (por ejemplo, jugando el fútbol o corriendo)? | ○ Muy frecuentemente<br>○ Frecuentemente<br>○ Ocasionalmente<br>○ Raramente |
| DEMactive3 | (Condicional) ¿Con qué frecuencia te molestan los mosquitos cuando estas afuera para actividades de ocio (p. ej. picnics, campamento, caminatas)? | ○ Muy frecuentemente<br>○ Frecuentemente<br>○ Ocasionalmente<br>○ Raramente |
| DEMactive4 | (Condicional) ¿Con qué frecuencia te molestan los mosquitos cuando estás trabajando? | ○ Muy frecuentemente<br>○ Frecuentemente<br>○ Ocasionalmente<br>○ Raramente |
| DEMactive5 | (Condicional) ¿Con qué frecuencia te molestan los mosquitos cuando estás durmiendo? | ○ Muy frecuentemente<br>○ Frecuentemente<br>○ Ocasionalmente<br>○ Raramente |
| DEMactive6 | (Condicional) ¿Con qué frecuencia le molestan los mosquitos cuando viajas por motivos educativos o laborales (trayectos hacía el trabajo)? | ○ Muy frecuentemente<br>○ Frecuentemente<br>○ Ocasionalmente<br>○ Raramente |

**Sección 2. Tu Comprensión de los Mosquitos y los Virus Transmitidos por Mosquitos**

Esta sección contiene declaraciones sobre la biología de los mosquitos, sus criaderos y los virus transmitidos por mosquitos.

<u>Las preguntas dentro de esta sección son aleatorias.</u>

| **Código de pregunta** | **Pregunta** | **Opción de respuesta** |
|---|---|---|
| KNbite | Los mosquitos solo pican a las personas durante el día. | ○ Sí<br>○ No<br>○ No lo sé. |
| KNbreed | En los jardines, los mosquitos pueden poner huevos en:<br><br>(Marque todo lo que corresponda)<br><br>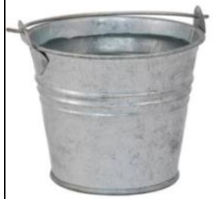 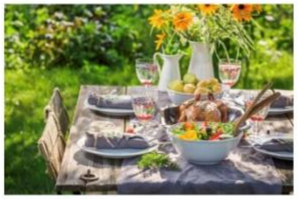 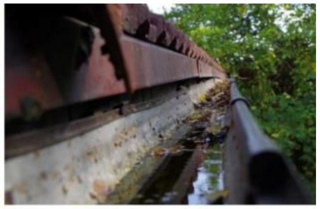<br>**A. Agua estancada en un cubo** **B. Sobras de comida en la mesa del jardín** **C. Agua estancada en canaletas** | ❑ A. Agua estancada en un cubo<br>❑ B. Sobras de comida en la mesa del jardín<br>❑ C. Agua estancada en canaletas<br>❑ Ninguna de las anteriores<br>❑ No lo sé. |

| | | |
|---|---|---|
| KNvirus | Los mosquitos son los principales transmisores de los siguientes virus:<br><br>(Marque todo lo que corresponda) | ❑ Virus del Nilo Occidental<br>❑ Virus del Chikungunya<br>❑ Virus del Zika<br>❑ Virus de la Influenza (Gripe)<br>❑ Virus del Dengue<br>❑ Virus de inmunodeficiencia humana (VIH)<br>❑ Virus del sarampión<br>❑ Ninguno de los anteriores<br>❑ No lo sé. |
| KNroute | Una persona puede contraer un virus transmitido por mosquito si:<br><br>(Marque todo lo que corresponda) | ❑ Recibe una picadura de un mosquito infectado<br>❑ Toca a una persona infectada<br>❑ Toca a un animal infectado<br>❑ Infectarse por el aire<br>❑ Tiene contacto sexual con una persona infectada<br>❑ Ninguno de los anteriores<br>❑ No lo sé. |

**Sección 3: Medidas de Prevención contra Mosquitos**

Para esta sección, responda las preguntas en relación con tus experiencias dentro y alrededor de tu casa. Todas las preguntas de esta sección están relacionadas con las medidas de prevención que utilizas contra los mosquitos.

Además, después de responder a las preguntas sobre posibles criaderos, nos gustaría darle más información sobre los criaderos de mosquitos.

Los mosquitos pueden poner huevos en aguas tranquilas. Por ejemplo, en un cubo o regadera en su jardín, agua estancada en canaletas obstruidas o en un barril de lluvia que se deja descubierto.

Los huevos de mosquito se convierten en mosquitos adultos en 14 días y necesitan agua para eso. Si eliminas el agua estancada todas las semanas, evitará que los huevos de mosquito se conviertan en mosquitos adultos. Esto puede reducir las poblaciones de mosquitos alrededor de tu casa.

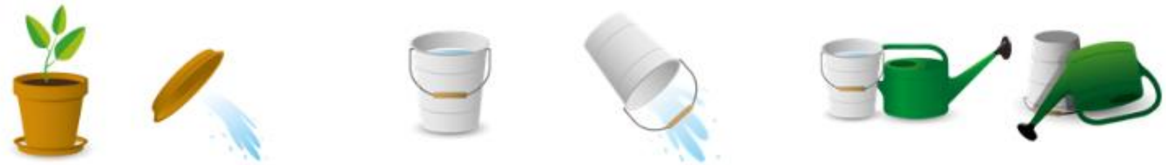

Fuente de imagen: NVWA muggen

| **Código de pregunta** | **Pregunta** | **Opción de respuesta** |
|---|---|---|
| PMuse | Selecciona todas las medidas de prevención que utilizas contra mosquitos: | ❑ Uso camisas de manga larga y |

| | | |
|---|---|---|
| | (Marque todo lo que corresponda) | pantalones largos > condicional. si se selecciona ir a PMreasonY<br>❑ Uso repelentes contra mosquitos para la piel (por ejemplo, DEET) > condicional. si se selecciona ir a PMreasonY<br>❑ Uso un zapper eléctrico > condicional. si se selecciona ir a PMreasonY<br>❑ Uso un ventilador eléctrico o aire acondicionado > condicional. si se selecciona ir a PMreasonY<br>❑ Uso un repelente de mosquitos enchufable en la toma de corriente > condicional. si se selecciona ir a PMreasonY<br>❑ Uso mosquiteras en puertas o ventanas<br>❑ Duermo con las ventanas del dormitorio cerradas > condicional. si se selecciona ir a PMreasonY<br>❑ Duermo con una mosquitera para cama > condicional. si se selecciona ir a PMreasonY<br>❑ Uso métodos naturales: ajo, hierbas, plantas > condicional. si se selecciona ir a PMreasonY<br>❑ Me deshago de aguas estancadas en mi casa o alrededores > condicional. si se selecciona ir a PMreasonY<br>❑ Otras > condicional. si se selecciona ir a PMreasonY |

| | | |
|---|---|---|
| | | ❑ Ninguna > condicional. si se selecciona ir a PMreasonN |
| PMreasonN | (Condicional)<br>¿Cuáles son las razones por las que no aplicas medidas de prevención contra mosquitos?<br>(Marque todo lo que corresponda) | ❑ No hay mosquitos donde yo vivo<br>❑ Las medidas de prevención son molestas<br>❑ Las medidas de prevención requieren tiempo<br>❑ Los mosquitos no me pican<br>❑ Las medidas de prevención son caras<br>❑ Otras |
| PMreasonY | (Condicional)<br>¿Cuáles son las razones por las que aplicas estas medidas de prevención contra mosquitos?<br>(Marque todo lo que corresponda) | ❑ Para reducir picaduras<br>❑ Para reducir el número de mosquitos dentro y alrededor de mi casa<br>❑ Para reducir la posibilidad de infectarme con virus transmitidos por mosquito<br>❑ Otras |
| RESpp | ¿Quién crees que es responsable de informar a la sociedad sobre medidas de prevención contra mosquitos?<br>(Marque todo lo que corresponda) | ❑ El Gobierno, incluyendo el Ministerio de Sanidad<br>❑ Los trabajadores sanitarios (como enfermeras y doctores)<br>❑ Los propios ciudadanos |
| RESpbreed | Pienso que soy el principal responsable de eliminar criaderos de mosquitos dentro y alrededor de mi casa. | ○ Muy de acuerdo<br>○ De acuerdo<br>○ Algo de acuerdo<br>○ Neutral<br>○ Algo en desacuerdo<br>○ En desacuerdo<br>○ Muy en desacuerdo |
| RESgovbreed | Pienso que el Gobierno* es el principal responsable de eliminar criaderos de mosquitos en mi barrio.*<br><br>* Pienso que el Gobierno* es el principal responsable de eliminar criaderos de mosquitos en mi barrio | ○ Muy de acuerdo<br>○ De acuerdo<br>○ Algo de acuerdo<br>○ Neutral<br>○ Algo en desacuerdo<br>○ En desacuerdo |

| | | |
|---|---|---|
| | | ○ Muy en desacuerdo |
| **Sección 4. Tu Opinión sobre las Medidas de Prevención**<br><br>En esta sección, las preguntas son sobre lo que crees sobre el uso de medidas de prevención y algunas medidas de prevención específicas (sobre las picaduras de mosquitos).<br><br><u>Las preguntas dentro de esta sección son aleatorias.</u> | | |
| **Código de pregunta** | **Pregunta** | **Opción de respuesta** |
| BENbites | El uso de repelentes para la piel (como DEET) previene las picaduras de mosquitos.<br>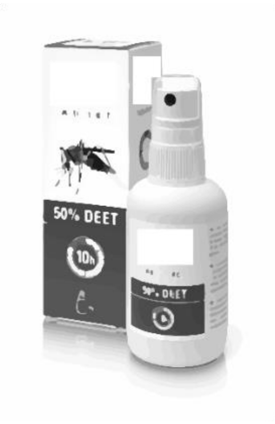 | ○ Muy de acuerdo<br>○ De acuerdo<br>○ Algo de acuerdo<br>○ Neutral<br>○ Algo en desacuerdo<br>○ En desacuerdo<br>○ Muy en desacuerdo |
| BENsafe | Los repelentes para la piel (como el DEET) son seguros de usar.<br>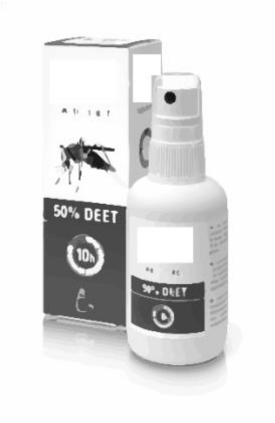 | ○ Muy de acuerdo<br>○ De acuerdo<br>○ Algo de acuerdo<br>○ Neutral<br>○ Algo en desacuerdo<br>○ En desacuerdo<br>○ Muy en desacuerdo |
| BENprev | Si utilizo medidas preventivas, evitaré que me piquen los mosquitos. | ○ Muy de acuerdo<br>○ De acuerdo<br>○ Algo de acuerdo<br>○ Neutral |

| | | |
|---|---|---|
| | | ○ Algo en desacuerdo<br>○ En desacuerdo<br>○ Muy en desacuerdo |
| BARannoy | Usar medidas de prevención contra los mosquitos es más irritantes que los mosquitos en sí. | ○ Muy de acuerdo<br>○ De acuerdo<br>○ Algo de acuerdo<br>○ Neutral<br>○ Algo en desacuerdo<br>○ En desacuerdo<br>○ Muy en desacuerdo |
| BARtime | Aplicar las medidas de prevención contra los mosquitos lleva demasiado tiempo. | ○ Muy de acuerdo<br>○ De acuerdo<br>○ Algo de acuerdo<br>○ Neutral<br>○ Algo en desacuerdo<br>○ En desacuerdo<br>○ Muy en desacuerdo |

**Sección 5. Mosquitos y Virus Transmitidos por Mosquitos**

Esta sección del cuestionario trata sobre tus experiencias dentro y alrededor de tu casa y en tu país de residencia. Nuestro objetivo es comprender cómo las molestias de los mosquitos, las medidas de prevención y los virus transmitidos por mosquitos afectan estas experiencias y tus puntos de vista sobre los mosquitos y los virus transmitidos por mosquitos.

Las preguntas dentro de esta sección son aleatorias.

| **Código de pregunta** | **Pregunta** | **Opción de respuesta** |
|---|---|---|
| SUSmosq | Vivo en un barrio donde hay muchos mosquitos. | ○ Muy de acuerdo<br>○ De acuerdo<br>○ Algo de acuerdo<br>○ Neutral<br>○ Algo en desacuerdo<br>○ En desacuerdo<br>○ Muy en desacuerdo |
| SUSbite | La probabilidad de ser picado por un mosquito en mi país de residencia es alta. | ○ Muy de acuerdo<br>○ De acuerdo<br>○ Algo de acuerdo<br>○ Neutral<br>○ Algo en desacuerdo<br>○ En desacuerdo<br>○ Muy en desacuerdo |
| SUSmbv | Me preocupa contraer una enfermedad causada por virus transmitido por mosquito en mi país de residencia. | ○ Muy de acuerdo<br>○ De acuerdo<br>○ Algo de acuerdo<br>○ Neutral<br>○ Algo en desacuerdo<br>○ En desacuerdo<br>○ Muy en desacuerdo |

| | | |
|---|---|---|
| SUSres | Estoy en riesgo de infectarme con un virus transmitido por mosquitos en mi país de residencia. | ○ Muy de acuerdo<br>○ De acuerdo<br>○ Algo de acuerdo<br>○ Neutral<br>○ Algo en desacuerdo<br>○ En desacuerdo<br>○ Muy en desacuerdo |
| SEVprobs | Enfermarse con un virus transmitido por mosquitos puede resultar en la hospitalización. | ○ Muy de acuerdo<br>○ De acuerdo<br>○ Algo de acuerdo<br>○ Neutral<br>○ Algo en desacuerdo<br>○ En desacuerdo<br>○ Muy en desacuerdo |
| SEVdeadly | Las personas pueden morir a causa de una infección por un virus transmitido por mosquitos. | ○ Muy de acuerdo<br>○ De acuerdo<br>○ Algo de acuerdo<br>○ Neutral<br>○ Algo en desacuerdo<br>○ En desacuerdo<br>○ Muy en desacuerdo |
| SEVqual | Al enfermarme a causa de un virus transmitido por mosquito, mi capacidad para realizar tareas diarias puede verse reducida. | ○ Muy de acuerdo<br>○ De acuerdo<br>○ Algo de acuerdo<br>○ Neutral<br>○ Algo en desacuerdo<br>○ En desacuerdo<br>○ Muy en desacuerdo |
| ControlA | Por favor, seleccione "Algo de acuerdo" como su opción de respuesta. | ○ Muy de acuerdo<br>○ De acuerdo<br>○ Algo de acuerdo<br>○ Neutral<br>○ Algo en desacuerdo<br>○ En desacuerdo<br>○ Muy en desacuerdo |
| **Sección 6. Concienzación sobre Medidas de Prevención**<br><br>Esta sección tiene como objetivo comprender tu confianza para picaduras y cómo le gustaría que se le recordara sobre el uso de medidas de prevención. Esta sección se refiere específicamente a tu en tu país de residencia.<br><br><u>Las preguntas dentro de esta sección son aleatorias.</u> | | |
| **Código de pregunta** | **Pregunta** | **Opción de respuesta** |
| SEbest | Sé que medidas de prevención son mejores contra las picaduras de mosquito. | ○ Muy de acuerdo<br>○ De acuerdo<br>○ Algo de acuerdo<br>○ Neutral<br>○ Algo en desacuerdo |

| | | |
|---|---|---|
| | | ○ En desacuerdo<br>○ Muy en desacuerdo |
| SEinfo | Sé dónde encontrar información sobre medidas de prevención contra las picaduras de mosquito. | ○ Muy de acuerdo<br>○ De acuerdo<br>○ Algo de acuerdo<br>○ Neutral<br>○ Algo en desacuerdo<br>○ En desacuerdo<br>○ Muy en desacuerdo |
| SEbreedid | Confío en que puedo identificar criaderos de mosquitos. | ○ Muy de acuerdo<br>○ De acuerdo<br>○ Algo de acuerdo<br>○ Neutral<br>○ Algo en desacuerdo<br>○ En desacuerdo<br>○ Muy en desacuerdo |
| SEbreedrem | Confío en que puedo eliminar criaderos de mosquitos dentro y alrededor de mi casa durante la temporada de mosquitos (de marzo a septiembre). | ○ Muy de acuerdo<br>○ De acuerdo<br>○ Algo de acuerdo<br>○ Neutral<br>○ Algo en desacuerdo<br>○ En desacuerdo<br>○ Muy en desacuerdo |
| CUEout | Durante el verano, estar en espacios exteriores (senderismo en la naturaleza, de acampada, de pícnic, al jardín) me recuerda que tengo que usar medidas preventivas contra mosquitos. | ○ Muy de acuerdo<br>○ De acuerdo<br>○ Algo de acuerdo<br>○ Neutral<br>○ Algo en desacuerdo<br>○ En desacuerdo<br>○ Muy en desacuerdo |
| CUEmosq | Los mosquitos dentro y alrededor de mi casa por la noche me recuerdan que debo usar medidas de prevención contra los mosquitos. | ○ Muy de acuerdo<br>○ De acuerdo<br>○ Algo de acuerdo<br>○ Neutral<br>○ Algo en desacuerdo<br>○ En desacuerdo<br>○ Muy en desacuerdo |
| CUEnotif | Recibir alertas de noticias sobre casos de virus transmitidos por mosquitos en mi área me recordaría usar medidas de prevención. | ○ Muy de acuerdo<br>○ De acuerdo<br>○ Algo de acuerdo<br>○ Neutral<br>○ Algo en desacuerdo<br>○ En desacuerdo<br>○ Muy en desacuerdo |
| ControlB | Por favor, seleccione "En desacuerdo" como su opción de respuesta. | ○ Muy de acuerdo<br>○ De acuerdo<br>○ Algo de acuerdo<br>○ Neutral<br>○ Algo en desacuerdo<br>○ En desacuerdo |

| | | |
|---|---|---|
| | | ○ Muy en desacuerdo |

**Sección 7. Información de Contexto**

En esta sección de la encuesta, hacemos algunas preguntas generales sobre lo que podría influir en tus pensamientos sobre los mosquitos y los virus transmitidos por mosquitos.

| **Código de pregunta** | **Pregunta** | **Opción de respuesta** |
|---|---|---|
| DEMmbvinf | Conozco a alguien que ha enfermado de infección por virus transmitido por mosquito. | ○ Sí<br>○ No<br>○ No lo sé. |
| DEMgp | Si tengo síntomas parecidos a los de una gripe, me pongo en contacto con mi médico de cabecera.*<br><br>**(Nos gustaría saber cuándo te pondrías en contacto con tu médico de cabecera. Esta pregunta no es específica para virus transmitidos por mosquito sino en general para situaciones en las que experimentas sintomatología de gripe, sin relación con la COVID-19)* | ○ Muy de acuerdo<br>○ De acuerdo<br>○ Algo de acuerdo<br>○ Neutral<br>○ Algo en desacuerdo<br>○ En desacuerdo<br>○ Muy en desacuerdo |
| DEMinfo | ¿Has leído u oído información sobre virus transmitidos por mosquito a través de alguna de las siguientes fuentes, recientemente?<br><br>(Marque todo lo que corresponda) | ❑ Profesionales sanitarios<br>❑ Página web del Gobierno<br>❑ Redes sociales (por ejemplo, Instagram, Twitter, Facebook, YouTube, artículos de prensa online)<br>❑ Familia y amigos<br>❑ Instituciones educativas (por ejemplo, escuelas o universidades)<br>❑ Páginas web institucionales (por ejemplo, OMS, ECDC, OIE, Ministerio de Sanidad)<br>❑ Televisión y medios de comunicación<br>❑ Periódico<br>❑ Radio<br>❑ Campañas de comunicación<br>❑ Ninguna de las anteriores |
| DEMsearch | ¿Dónde le gustaría encontrar o recibir información sobre mosquitos y virus transmitidos por mosquitos?<br><br>(Marque todo lo que corresponda) | ❑ Profesionales sanitarios<br>❑ Página web del Gobierno<br>❑ Redes sociales (por ejemplo, Instagram, Twitter, Facebook, YouTube, artículos de prensa online) |

| | | |
|---|---|---|
| | | ❑ Familia y amigos<br>❑ Instituciones educativas (por ejemplo, escuelas o universidades)<br>❑ Páginas web institucionales (por ejemplo, OMS, ECDC, OIE, Ministerio de Sanidad)<br>❑ Televisión y medios de comunicación<br>❑ Periódico<br>❑ Radio<br>❑ Campañas de comunicación<br>❑ Ninguna de las anteriores<br>❑ Otro [ respuesta abierta ] |
| **Sección 8. Historia de Viajes**<br><br>Esta sección trata sobre tu historial de viajes. | | |
| **Código de pregunta** | **Pregunta** | **Opción de respuesta** |
| DEMtravel | ¿Dónde has estado de vacaciones en los últimos dos años (2020-2022)?<br><br>(Marque todo lo que corresponda) | ❑ No he salido de mi país de residencia. > Condicional. si se selecciona ir a DEMadvice<br>❑ He viajado dentro de Europa.> Condicional. si se selecciona ir a DEMeu<br>❑ He viajado fuera de Europa. > Condicional. si se selecciona ir a DEMnoneu |
| DEMeu | (Condicional)<br><br>Por favor, selecciona qué regiones y países europeos has visitado durante tus vacaciones en los últimos 2 años (2020-2022).<br><br>(Marque todo lo que corresponda) | ❑ Austria<br>❑ Alemania<br>❑ Bélgica<br>❑ Dinamarca<br>❑ Este de Europa (Hungría, República Checa, Eslovenia, Polonia, Croacia)<br>❑ Finlandia<br>❑ Francia (incluye Córcega)<br>❑ Grecia e islas griegas<br>❑ Holanda/Países Bajos<br>❑ Irlanda<br>❑ Islandia |

| | | |
|---|---|---|
| | | ❑ Islas Canarias, Azores, Madeira<br>❑ Italia (incluye Sicilia, Cerdeña)<br>❑ Luxemburgo<br>❑ Noruega<br>❑ Portugal<br>❑ Suecia<br>❑ Swiza<br>❑ Turquía<br>❑ Reino Unido (Inglaterra, Escocia, Gales, Irlanda del Norte)<br>❑ Otro: [respuesta abierta] |
| DEMnoneu | (Condicional) ¿Qué regiones fuera de Europa has visitado durante tus vacaciones en los últimos 2 años (2020-2022)?<br><br>*Ver mapa de las regiones debajo.<br><br>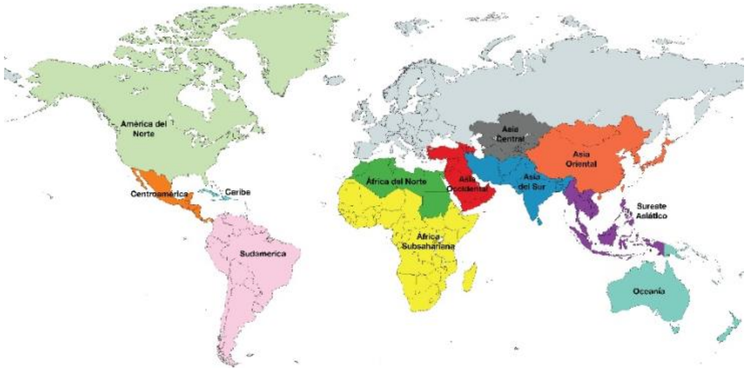 | ❑ África del Norte<br>❑ África Subsahariana (incluye África del Oeste, Centro, Este y Sur)<br>❑ América del Norte<br>❑ Caribe<br>❑ Centroamérica<br>❑ Sudamérica<br>❑ Asia Central<br>❑ Asia Oriental<br>❑ Sureste Asiático<br>❑ Asia del Sur<br>❑ Asia Occidental<br>❑ Oceanía |
| DEMadvice | ¿Alguna vez has recibido recomendaciones de salud para viajar relacionadas con virus transmitidos por mosquito?<br><br>* La malaria es una enfermedad parasitario transmitido por mosquitos. | ❑ Sí<br>❑ No<br>❑ No estoy seguro/a de haber recibido recomendaciones de salud para viajar relacionadas con virus transmitidos por mosquito.<br>❑ No, pero he recibido consejos de salud relacionados con viajes sobre la malaria.* |
| **Sección 9. Demografía**<br><br>En esta sección final de la encuesta, hacemos algunas preguntas generales sobre tus características demográficas. | | |

| | | |
|---|---|---|
| **Código de pregunta** | **Pregunta** | **Opción de respuesta** |
| DEMage | ¿Cuantos años tienes? | Respuesta numérica abierta |
| DEMgender | ¿Cuál es tu género? | ○ Hombre<br>○ Mujer<br>○ Otro [Respuesta abierta]<br>○ Prefiero no contestar |
| DEMeduc | ¿Cuál es el grado o nivel de educación más alto que ha completado? | ○ Escuela primaria<br>○ Escuela secundaria<br>○ Postsecundaria (incluye colegio técnico/comunitario y universidad)<br>○ Ninguna de las anteriores |
| DEMoccustatus | ¿Cuál es su situación laboral actual? | ○ Empleado > Condicional. si se selecciona ir a DEMindustry<br>○ Estudiante > Condicional. si se selecciona ir a DEMindustry<br>○ Amo/a de casa<br>○ (Actualmente) desempleado/a<br>○ Jubilado |
| DEMindustry | (Condicional) ¿Cuál de las siguientes categorías describe mejor la industria en la que trabaja o estudia principalmente (independientemente de su puesto actual)? | ❑ Arquitectura, Ingeniería, Informática y Matemáticas<br>❑ Ejército y Cuerpos Policiales<br>❑ Arte, Diseño, Ocio, Deporte y Medios de comunicación<br>❑ Construcción y Extracción, Edificación, Mantenimiento y Limpieza de recintos<br>❑ Educación, Entrenamiento, Bibliotecas, Servicios Sociales y Comunitarios<br>❑ Agricultura, Ganadería, Pesca, Servicios Forestales |

| | | |
|---|---|---|
| | | ❑ Medicina, Sanidad<br>❑ Fontanería, Carpintería, Artesanía y relacionados<br>❑ Servicios legales, Empresariado y Finanzas<br>❑ Administración<br>❑ Técnicos Científicos y Profesionales<br>❑ Servicios, Ventas, Hostelería y relacionados (por ejemplo, procesamiento de alimentos, cuidados personales y mercado inmobiliario)<br>❑ Movilidad, Transporte |
| DEMres | ¿Cuál es tu país de residencia? | ○ España (incluye Islas Canarias, Islas Baleares, Ceuta y Melilla)<br>○ Otro [ respuesta abierta ] |
| DEMarea | ¿Vives en una zona urbana o rural? | ○ Urbana<br>○ Rural<br>○ No lo sé. |
| DEMprovince | Por favor, especifica tu provincia. | La lista desplegable |
| DEMcs | ¿Has utilizado alguna vez alguno de los siguientes sitios web (ciencia ciudadana) o aplicaciones móviles?<br><br>(Marque todo lo que corresponda) | ❑ Mosquito Alert<br>❑ Muggenradar<br>❑ Mückenatlas<br>❑ ZanzaMapp<br>❑ No he usado ninguno de las siguientes páginas web o aplicaciones de ciencia ciudadana. |
| Encuesta termina | | |
