# Supplementary material for: Development and validation of the MosquitoWise survey to assess perceptions towards mosquitoes and mosquito-borne viruses in Europe
Source: Sci Rep. 2024 Jan 20;14:1777. doi: 10.1038/s41598-024-52219-9 (PMC10799950; doi:10.1038/s41598-024-52219-9)
Supplement: Supplementary file 3 — Supplementary Table 1. [file 41598_2024_52219_MOESM3_ESM.pdf]

Supplementary Table 1. Factor loadings results after distribution of Survey Version 1 in the United Kingdom (Sample 1).

Question Code	Question	Factor Loadings (Survey Version 1)					
		Model A	Model B	Model C	Model D	Model E	Model F
SUSbite	I am worried about getting bitten by a mosquito in my country of residence	0.058	0.665	0.666	0.552	0.663	0.666
SUSres	If I get bitten by a mosquito in my country of residence, I might get sick from a mosquito-borne virus.	0.119	0.440	0.440	0.808	0.429	0.429
SUStravel1	I think it is possible to get infected with a mosquito-borne virus while: I am travelling within Europe.	0.437	NA	NA	NA	NA	NA
SUStravel2	I think it is possible to get infected with a mosquito-borne virus while: I am travelling in Thailand.	0.758	NA	NA	NA	NA	NA
SUStravel3	I think it is possible to get infected with a mosquito-borne virus while: I am travelling in South Africa.	0.765	NA	NA	NA	NA	NA
SUStravel4	I think it is possible to get infected with a mosquito-borne virus while: I am travelling in Brazil.	0.780	NA	NA	NA	NA	NA
SUStravel5	I think it is possible to get infected with a mosquito-borne virus while: I am travelling in the United States.	0.555	NA	NA	NA	NA	NA

SUSmbv	I am worried about getting sick from a mosquito-borne virus in my country of residence.	0.052	0.838	0.879	0.302	0.854	0.849
SEVprobs	Getting sick with a mosquito-borne virus can cause serious health problems.	0.860	0.854	0.837	0.227	0.867	0.853
SEVdeadly	A mosquito-borne virus can be deadly for humans.	0.785	0.799	0.818	0.328	0.824	0.836
SEVqual	Becoming sick from a mosquito-borne virus can change a person's quality of life.	0.860	0.859	0.843	0.288	0.853	0.860
SEVtreat	A mosquito-borne virus is hard to treat.	0.452	0.438	0.393	0.848	0.388	0.383
SEVrecover	It is easy to recover from a mosquito-borne virus.	-0.353	-0.364	NA	NA	NA	NA
BBbites	I think skin repellents (such as deet) prevent mosquito bites	0.457	0.415	0.419	0.827	0.418	0.488
BBskin	I think that mosquito repellents (such as deet) applied to the skin are safe to use	0.236	0.226	0.234	0.947	0.230	0.322

BBcost	I think skin repellents (such as deet) are too expensive.	0.245	0.280	0.271	0.926	0.284	0.263
BBbreedbite	If I remove mosquito breeding sites, I will reduce the chance I get bitten.	0.705	0.714	0.718	0.480	0.707	0.639
CUErecom	Recommendations on preventive measures against mosquitoes bites would encourage me to use them.	0.405	0.608	0.619	0.593	0.717	0.729
CUEremind	In my country of residence, I would use preventive practices if the government reminds me	0.336	0.564	0.565	0.678	0.639	0.629
CUEnotif	If I get a notification about mosquito-borne virus cases in my area, I would use preventive measures.	0.618	0.396	0.390	0.861	NA	NA
CUEdoc	My doctor/nurse impacts my decision to use preventive measures against mosquitoes while travelling.	0.225	0.176	0.171	NA	NA	NA
SEbreedid	I know how to identify mosquito breeding sites.	0.546	0.600	0.581	0.658	0.604	NA
SEbreedrem	During mosquito season (March through September), I can remove mosquito breeding sites in and around my house.	0.576	0.639	0.626	0.602	0.643	0.362

SEpmuse	It would be easy for me to keep using preventive measures throughout mosquito season.	0.289	0.298	0.305	0.915	0.282	0.297
SEinfo	I can search for information about using preventive measures against mosquitoes.	0.343	0.284	0.294	0.907	0.295	0.327

Items with factor loadings < 0.3 were removed from the survey.
