# Supplementary material for: Development and validation of the MosquitoWise survey to assess perceptions towards mosquitoes and mosquito-borne viruses in Europe
Source: Sci Rep. 2024 Jan 20;14:1777. doi: 10.1038/s41598-024-52219-9 (PMC10799950; doi:10.1038/s41598-024-52219-9)
Supplement: Supplementary file 2 — Supplementary Figure 1. [file 41598_2024_52219_MOESM2_ESM.pdf]

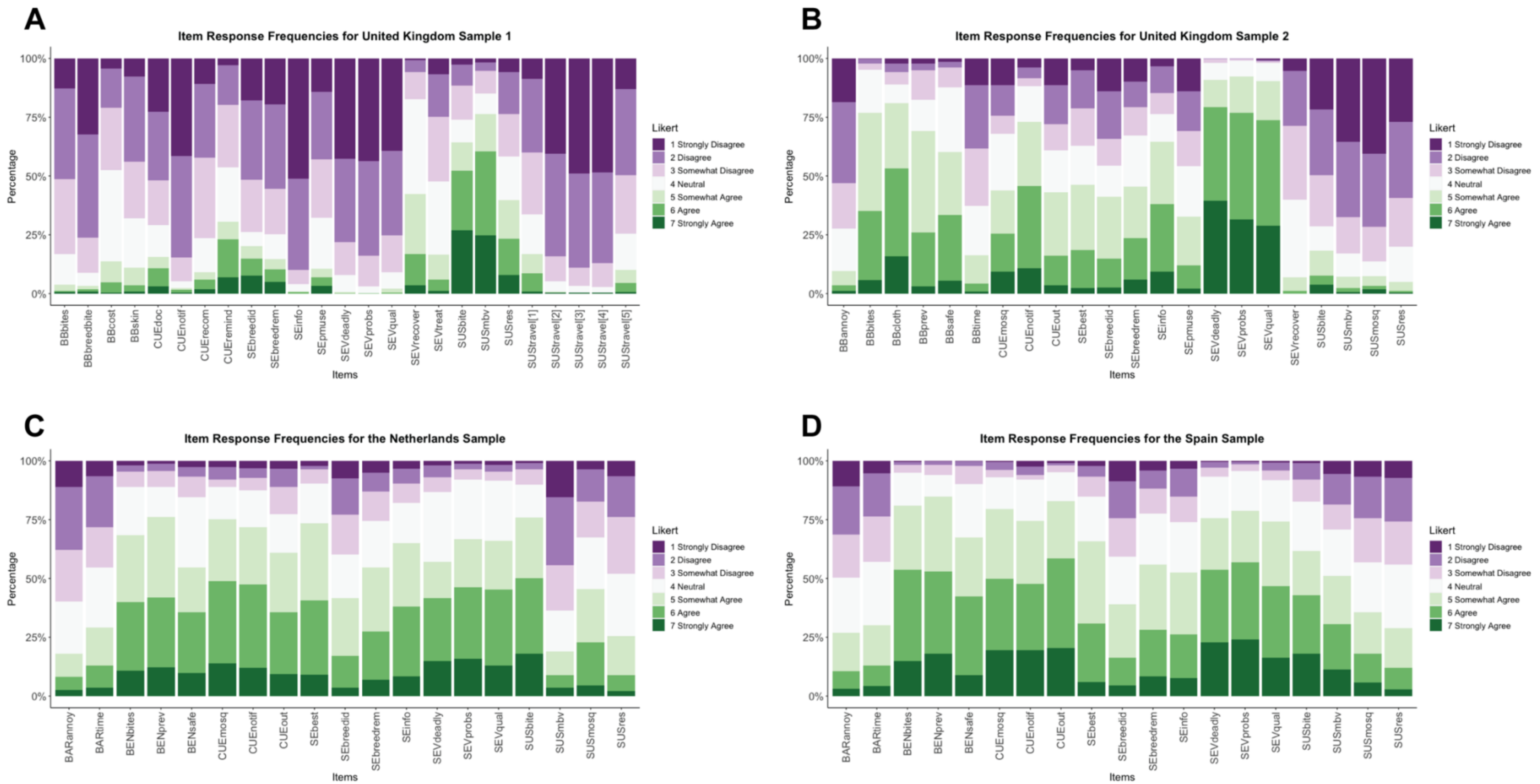

Supplementary Figure 1. Item Response frequency graphs of all survey versions for Health Belief Model items. **A)** Item response frequency graph of Survey Version 1 responses from United Kingdom Sample 1. **B)** Item response frequency graph of Survey Version 2 responses from United Kingdom Sample 2. **C)** Item response frequency graph of Survey Version 3 responses from the Netherlands Sample. **D)** Item response frequency graph of Survey Version 3 responses from the Spain Sample.
